# Supplementary material for: Facile conversion of water to functional molecules and cross-linked polymeric films with efficient clusteroluminescence
Source: Nat Commun. 2023 May 30;14:3115. doi: 10.1038/s41467-023-38769-y (PMC10229605; doi:10.1038/s41467-023-38769-y)
Supplement: Supplementary file 1 — Supplementary Information [file 41467_2023_38769_MOESM1_ESM.pdf]

Supplementary Information

Facile conversion of water to functional molecules and cross-linked polymeric films with efficient clusteroluminescence

Bo Song,[1] Jianyu Zhang,[1] Jiadong Zhou,[2] Anjun Qin,[2,3] Jacky W. Y. Lam,[1]* Ben Zhong Tang[1,3,4]*

[1] Department of Chemistry, Hong Kong Branch of Chinese National Engineering Research Center for Tissue Restoration and Reconstruction, and Guangdong-Hong Kong-Macau Joint Laboratory of Optoelectronic and Magnetic Functional Materials, The Hong Kong University of Science and Technology, Clear Water Bay, Kowloon, Hong Kong, 999077, China

[2] State Key Laboratory of Luminescent Materials and Devices, Guangdong Provincial Key Laboratory of Luminescence from Molecular Aggregates, South China University of Technology, Guangzhou 510640, China

[3] Center for Aggregation-Induced Emission, AIE Institute, South China University of Technology, Guangzhou 510640, China

[4] Shenzhen Institute of Aggregate Science and Technology, School of Science and Engineering, The Chinese University of Hong Kong, Shenzhen, Guangdong 518172, China.

Contents

Structural characterization and photophysical properties of small molecules	S3
Synthetic procedure and characterization data for polymer PTMP	S23
Photophysical properties of polymer PTMP	S25
Additional data	S30

Structural characterization and photophysical properties of small molecules

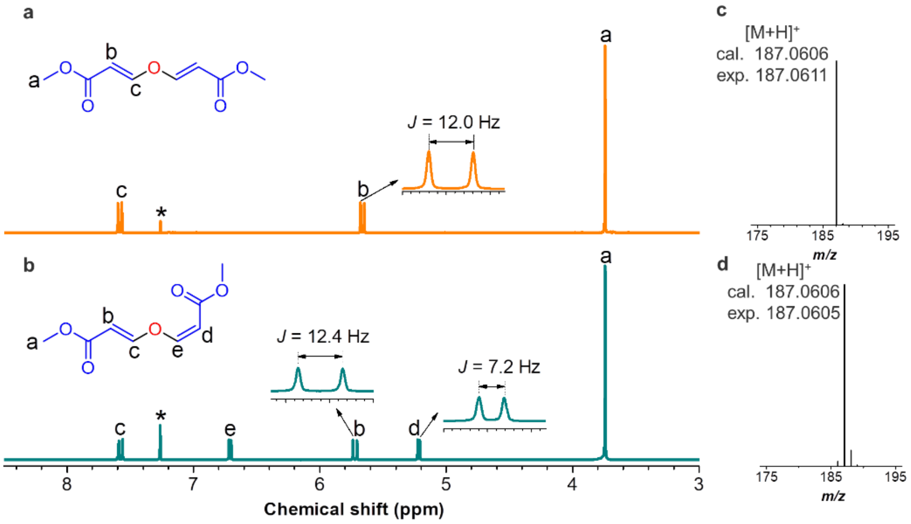

Supplementary Figure 1. (a, b) ^{1}H NMR spectra of (a) *EE*-DMODA, and (b) *EZ*-DMODA in $CDCl_3$. (c, d) HRMS of (c) *EE*-DMODA, and (d) *EZ*-DMODA.

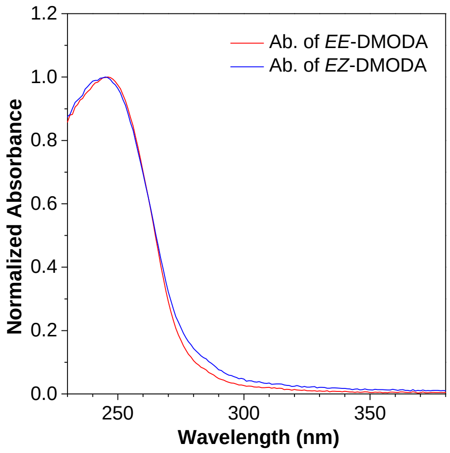

Supplementary Figure 2. Absorption spectra of *EE*-DMODA and *EZ*-DMODA in THF solution, concentration = 1×10^{-4} M.

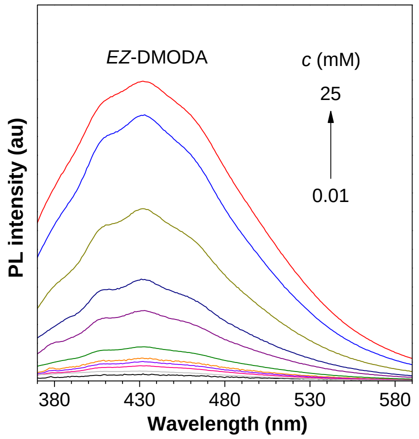

Supplementary Figure 3. Photoluminescence spectra of *EZ*-DMODA in THF with different concentrations (λ_{ex} = 330 nm).

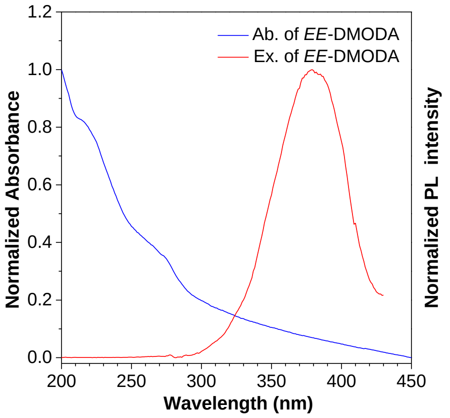

Supplementary Figure 4. Absorption and excitation spectra of *EE*-DMODA in solid (λ_{em} = 445 nm).

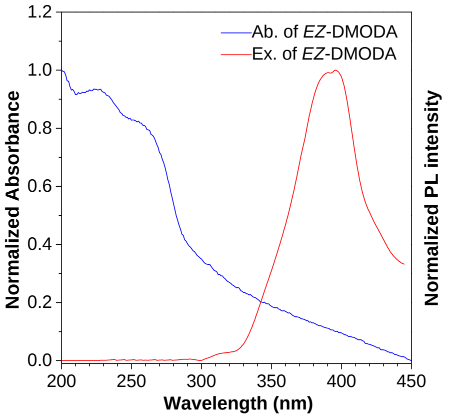

Supplementary Figure 5. Absorption and excitation spectra of *EZ*-DMODA in solid (λ_{em} = 478 nm).

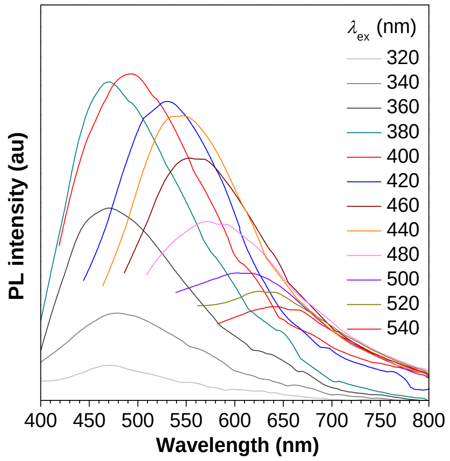

Supplementary Figure 6. Photoluminescence spectra of *EZ*-DMODA in solid.

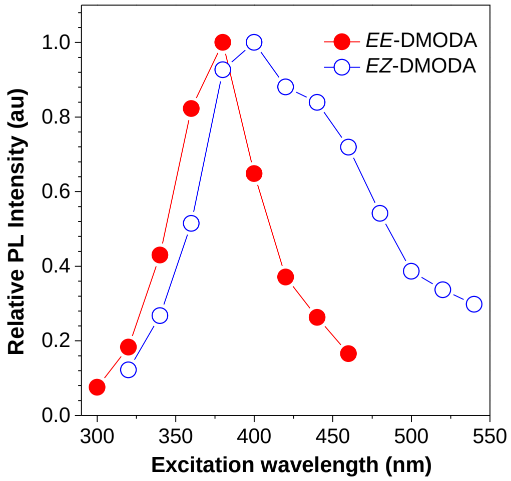

Supplementary Figure 7. Plots of relative photoluminescence intensities *versus* excitation wavelength.

Supplementary Table 1. Photophysical properties of clusteroluminogens.

	λ_{em} (nm)[a]	Φ (%)[b]	τ_{avg} (ns)[c]
EE-DMODA	445	12.9	3.7
EZ-DMODA	478	4.5	2.1
EE-DMODA-^{18}O	485	20.6	3.5
EZ-DMODA-^{18}O	493	10.4	1.8
EE-DMODA-D_4	437	6.5	3.0
EZ-DMODA-D_4	452	3.1	1.9
EE-OBBO	504	16.7	3.5

[a] λ_{em} = emission maximum in the solid state (λ_{ex} = 380 nm). [b] Absolute fluorescence quantum yield, measured by an integrating sphere. [c] The amplitude-weighted lifetime in the solid state (λ_{ex} = 365 nm). They were calculated from $\tau_{avg} = \tau_1 * Rel_1\% + \tau_2 * Rel_2\% + \tau_3 * Rel_3\%$.

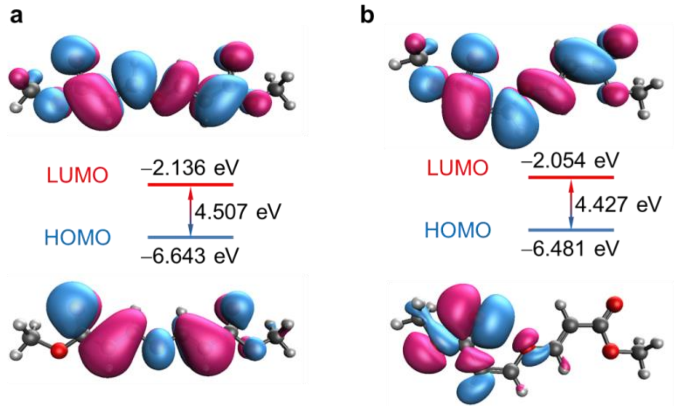

Supplementary Figure 8. Frontier molecular orbitals of (a) *EE*-DMODA and (b) *EZ*-DMODA based on the optimized excited-state geometries.

Supplementary Table 2. Single-crystal data of *EE*-DMODA, *EZ*-DMODA, *EE*-DMODA-^{18}O, and *EE*-OBBO.

Compound	*EE*-DMODA	*EZ*-DMODA	*EE*-DMODA-^{18}O	*EE*-OBBO
CCDC number	2177902	2177911	2177917	2177919
Empirical formula	$C_8H_{10}O_5$	$C_8H_{10}O_5$	$C_8H_{10}O_5$	$C_8H_{10}O_3$
Formula weight	186.16	186.16	186.16	154.16
Temperature/K	150.00(10)	149.99(10)	170(2)	149.99(10)
Space group	P21/n	P21/n	P21/n	P-1
a/Å	3.8457(4)	3.8795(19)	3.8197(2)	3.8800(3)
b/Å	30.207(3)	27.108(11)	30.1413(18)	9.2102(5)
c/Å	7.7596(7)	8.524(3)	7.7184(4)	11.2952(6)
α/ °	90	90	90	91.443(4)
β/ °	101.215(10)	102.02(5)	101.768(2)	95.625(5)
γ/ °	90	90	90	101.351(6)
Volume/Å^3	884.19(15)	876.8(6)	869.95(8)	393.43(4)
Z	4	4	4	2
Density/ g·cm^{-3}	1.398	1.410	1.421	1.301
F(000)	392	392	392	164
h_{max}, k_{max}, l_{max}	3, 36, 9	3, 27, 10	4, 38, 8	2, 11, 13
Goodness-of-fit on F^2	1.038	1.205	1.108	1.074
R_1 [I>=2σ (I)]	0.0629	0.1874[a]	0.0448	0.0508
wR_2 [I>=2σ (I)]	0.1758	0.4211[a]	0.1149	0.1417
R_1 [all data]	0.0751	0.2343[a]	0.0484	0.0557
wR_2 [all data]	0.1888	0.4603[a]	0.1188	0.1477

[a] We tried our best to grow better single crystal, but the plate crystals always caused the lack of high I/σ diffracted signals at higher θ region, leading to the high R_1 and wR_2 values.

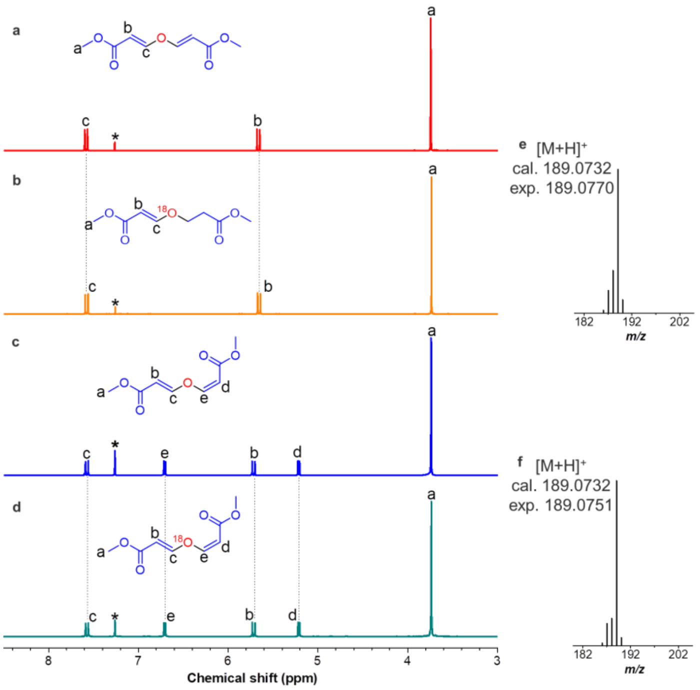

Supplementary Figure 9. (a-d) ^{1}H NMR spectra of (a) *EE*-DMODA, (b) *EE*-DMODA-^{18}O, (c) *EZ*-DMODA, and (d) *EZ*-DMODA-^{18}O in $CDCl_3$, (e, f) HRMS of (e) *EE*-DMODA-^{18}O, and (f) *EZ*-DMODA-^{18}O.

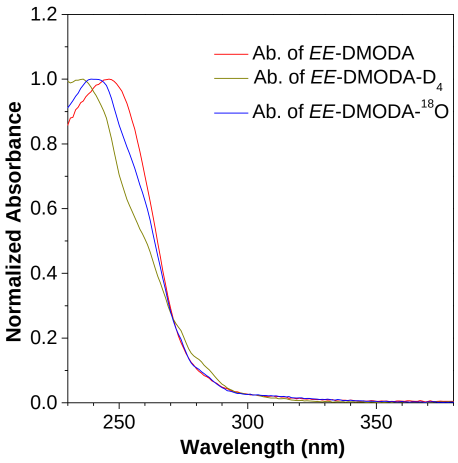

Supplementary Figure 10. Absorption spectra of *EE*-DMODA, *EE*-DMODA-D_4, and *EE*-DMODA-^{18}O in THF, concentration = 1×10^{-4} M.

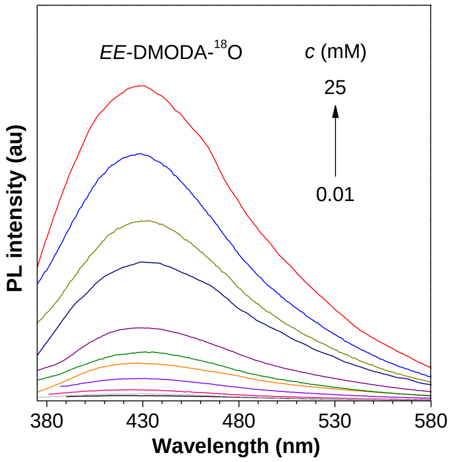

Supplementary Figure 11. Photoluminescence spectra of *EE*-DMODA-^{18}O in THF with different concentrations (λ_{ex} = 330 nm).

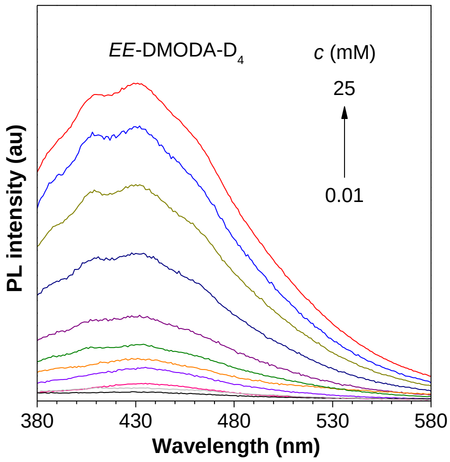

Supplementary Figure 12. Photoluminescence spectra of *EE*-DMODA-D_4 in THF with different concentrations (λ_{ex} = 330 nm).

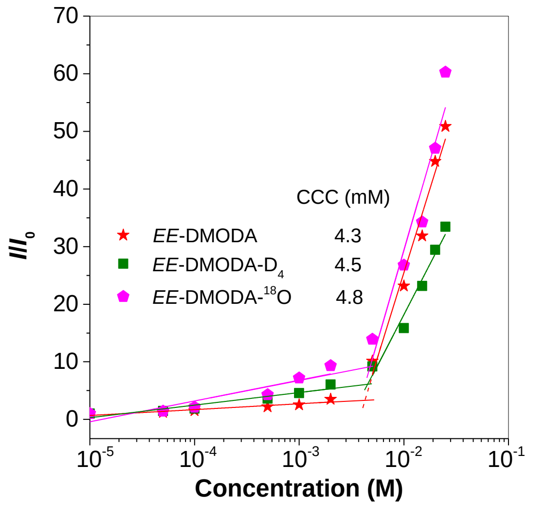

Supplementary Figure 13. Plots of relative photoluminescence intensity (I/I_0) versus concentration, I_0 = intensity at 10^{-5} M.

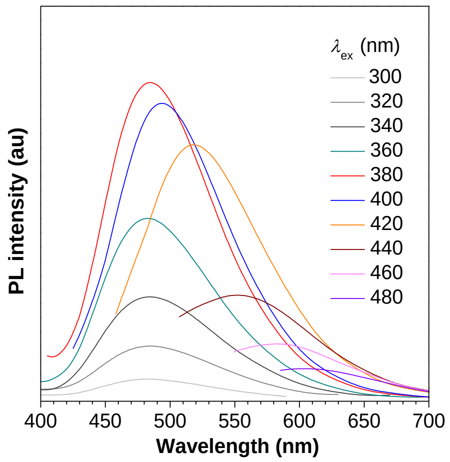

Supplementary Figure 14. Photoluminescence spectra of *EE*-DMODA-^{18}O in solid.

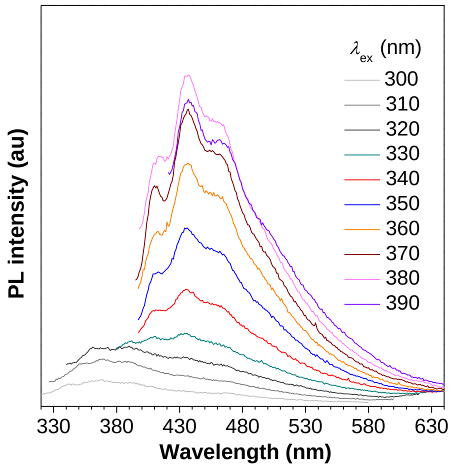

Supplementary Figure 15. Photoluminescence spectra of *EE*-DMODA-D_4 in solid.

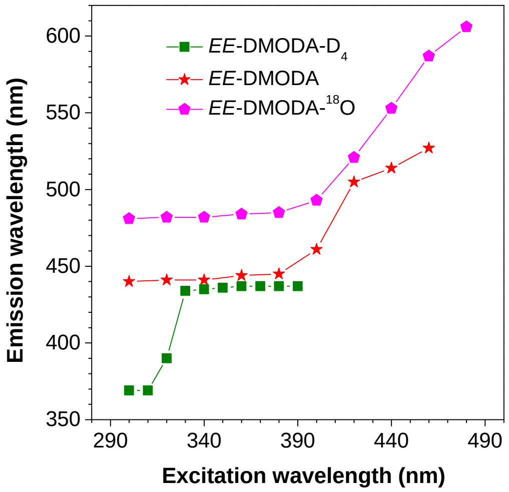

Supplementary Figure 16. Plots of emission wavelengths *versus* excitation wavelength.

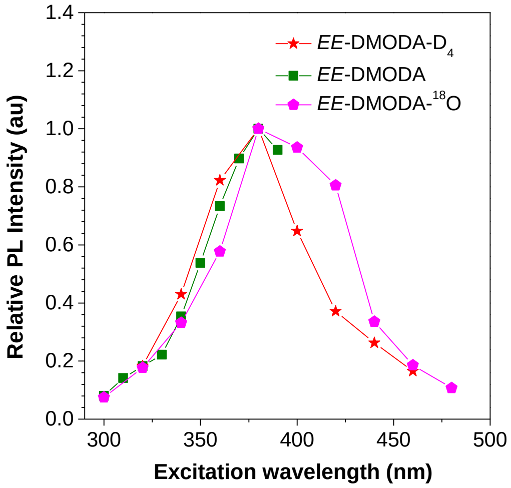

Supplementary Figure 17. Plots of relative photoluminescence intensities *versus* excitation wavelength.

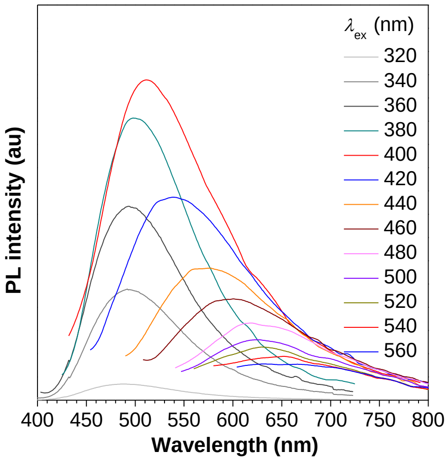

Supplementary Figure 18. Photoluminescence spectra of *EZ*-DMODA-^{18}O in solid.

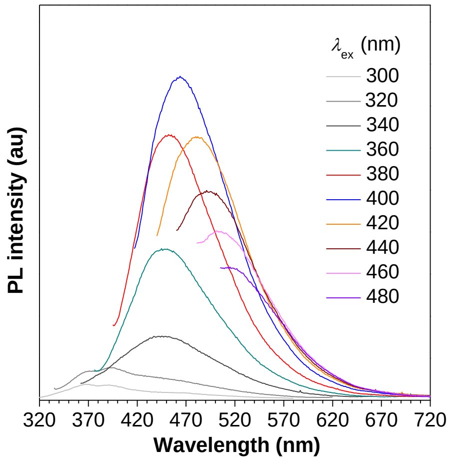

Supplementary Figure 19. Photoluminescence spectra of *EZ*-DMODA-D_4 in solid.

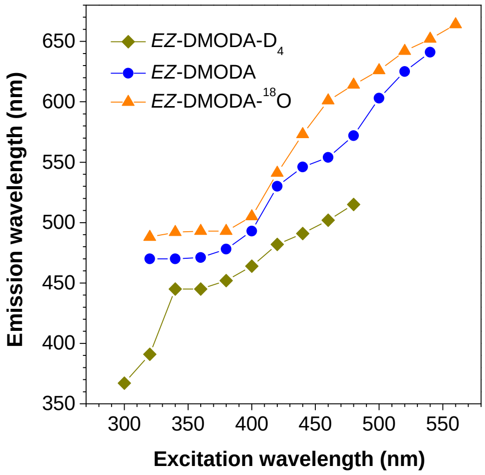

Supplementary Figure 20. Plots of emission wavelengths *versus* excitation wavelength.

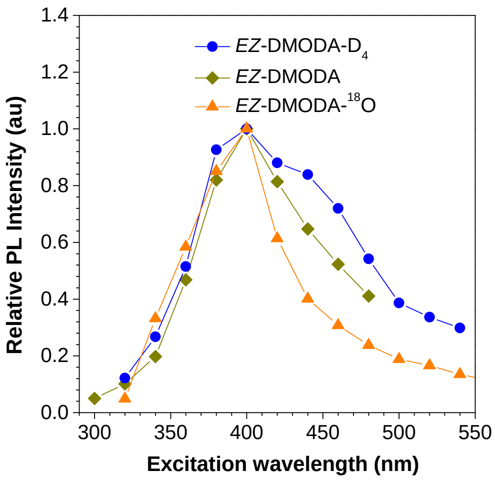

Supplementary Figure 21. Plots of relative photoluminescence intensities *versus* excitation wavelength.

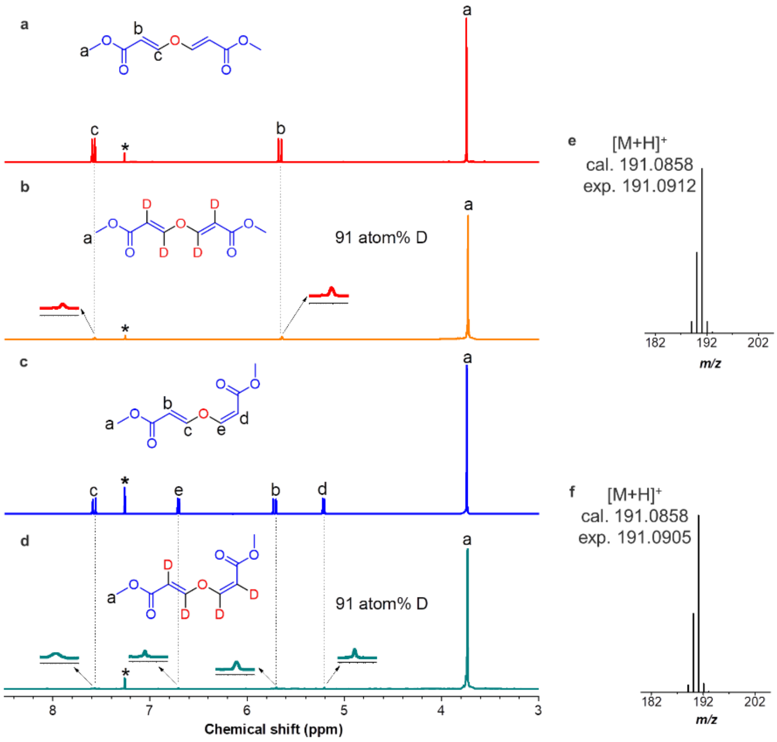

Supplementary Figure 22. (a-d) ^{1}H NMR spectra of (a) *EE*-DMODA, (b) *EE*-DMODA-D$_4$, (c) *EZ*-DMODA, and (d) *EZ*-DMODA-D$_4$ in CDCl$_3$. (e, f) HRMS of (e) *EE*-DMODA-D$_4$, and (f) *EZ*-DMODA-D$_4$.

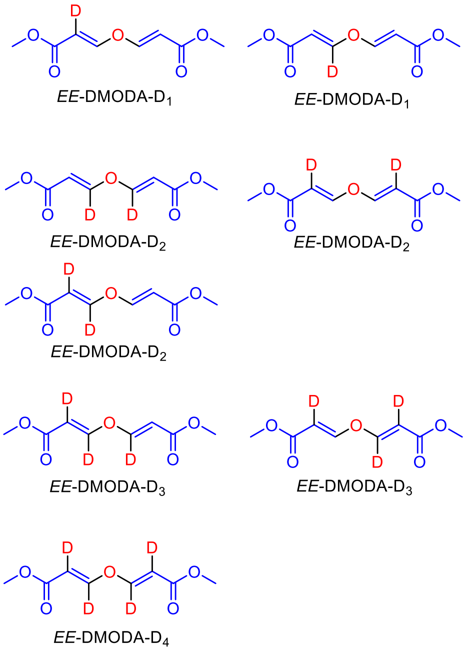

Supplementary Figure 23. Different compounds due to incomplete deuterated efficiency.

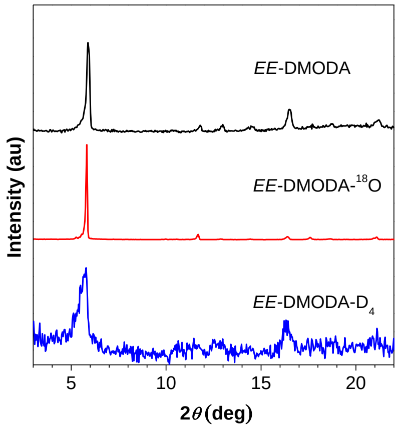

Supplementary Figure 24. PXRD patterns of *EE*-DMODA, *EE*-DMODA-D_4 and *EE*-DMODA-^{18}O.

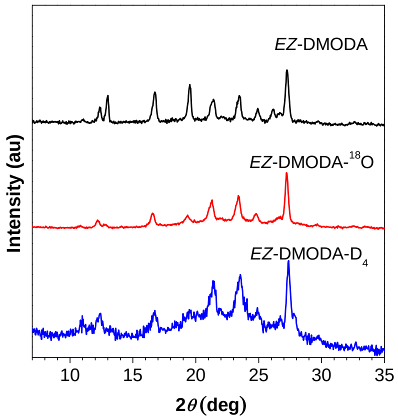

Supplementary Figure 25. PXRD patterns of *EZ*-DMODA, *EZ*-DMODA-D_4 and *EZ*-DMODA-^{18}O.

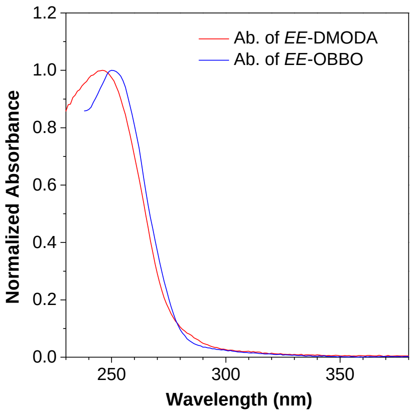

Supplementary Figure 26. Absorption spectra of *EE*-DMODA and *EE*-OBBO in THF, concentration = 1×10^{-4} M.

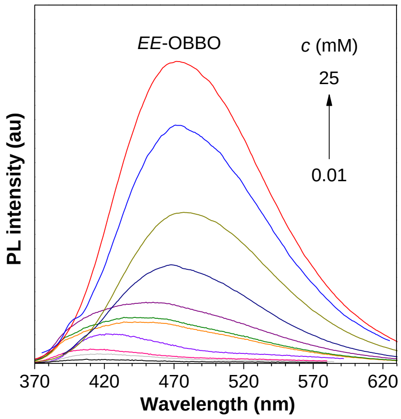

Supplementary Figure 27. Photoluminescence spectra of *EE*-OBBO in THF with different concentrations (λ_{ex} = 330 nm).

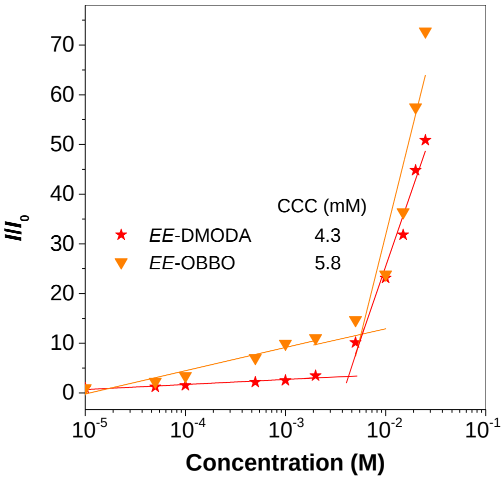

Supplementary Figure 28. Plots of relative photoluminescence intensity (I/I_0) versus concentration, I_0 = intensity at 10^{-5} M.

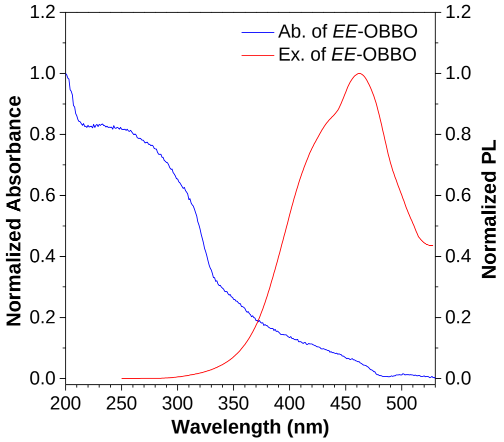

Supplementary Figure 29. Absorption and excitation spectra of *EE*-OBBO in solid (λ_{em} = 550 nm).

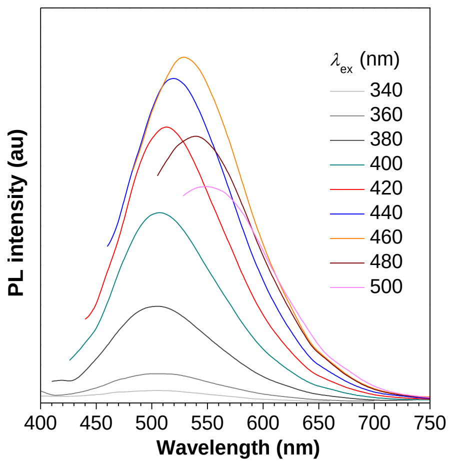

Supplementary Figure 30. Photoluminescence spectra of *EE*-OBBO in solid.

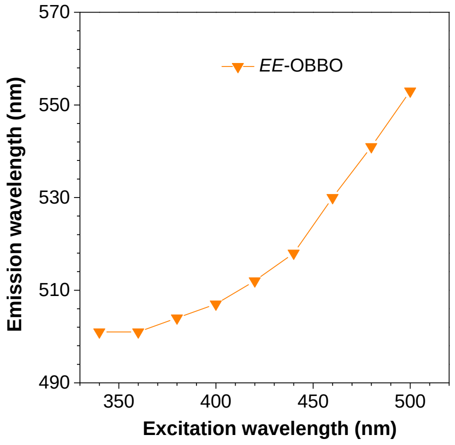

Supplementary Figure 31. Plots of emission wavelengths *versus* excitation wavelength.

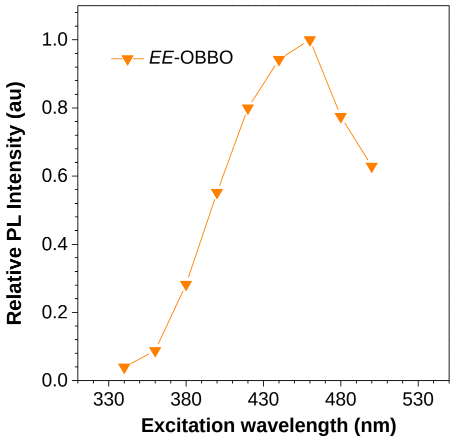

Supplementary Figure 32. Plots of relative photoluminescence intensities *versus* excitation wavelength.

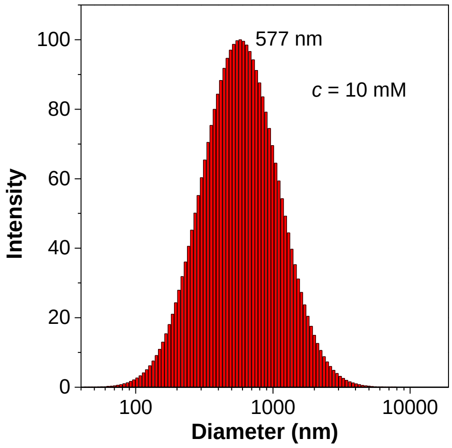

Supplementary Figure 33. Dynamic light scattering diagram of *EE*-OBBO in THF, [*EE*-OBBO] = 10 mM.

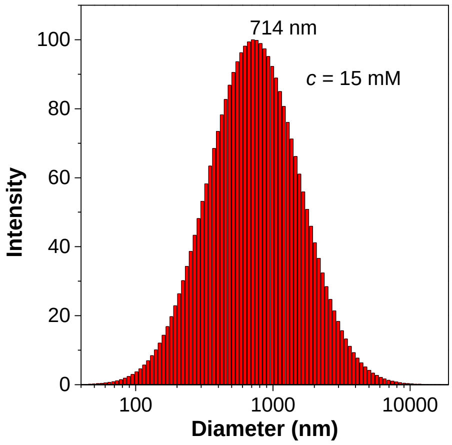

Supplementary Figure 34. Dynamic light scattering diagram of *EE*-OBBO in THF, [*EE*-OBBO] = 15 mM.

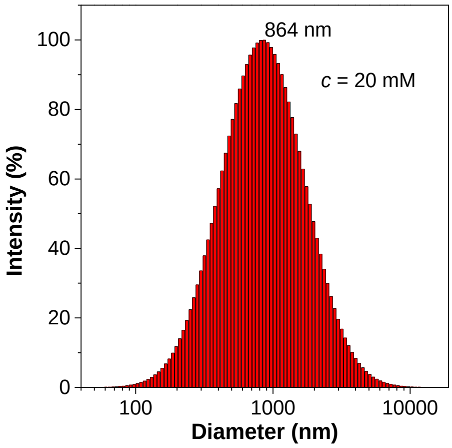

Supplementary Figure 35. Dynamic light scattering diagram of *EE*-OBBO in THF, [*EE*-OBBO] = 20 mM.

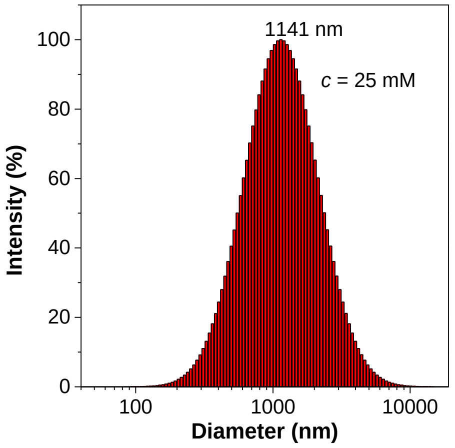

Supplementary Figure 36. Dynamic light scattering diagram of *EE*-OBBO in THF, [*EE*-OBBO] = 25 mM.

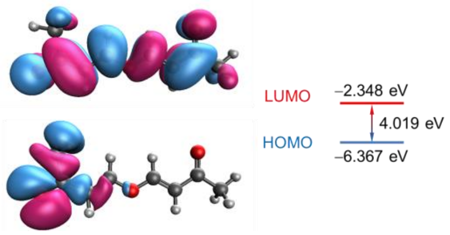

Supplementary Figure 37. Frontier molecular orbitals of *EE*-OBBO based on the optimized excited-state geometry.

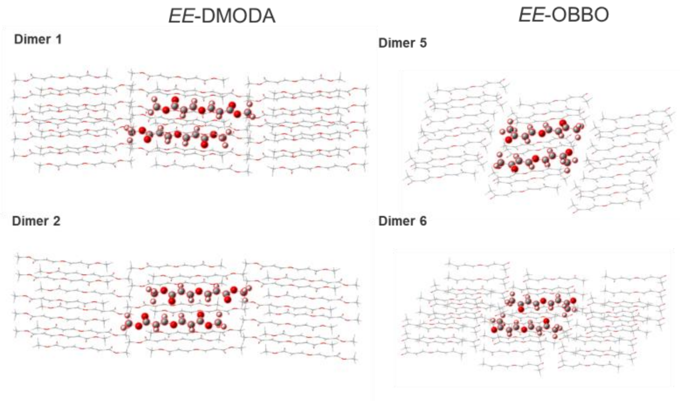

Supplementary Figure 38. Setup of QM/MM models for theoretical calculation of crystal phase, which were extracted from crystal packing structures.

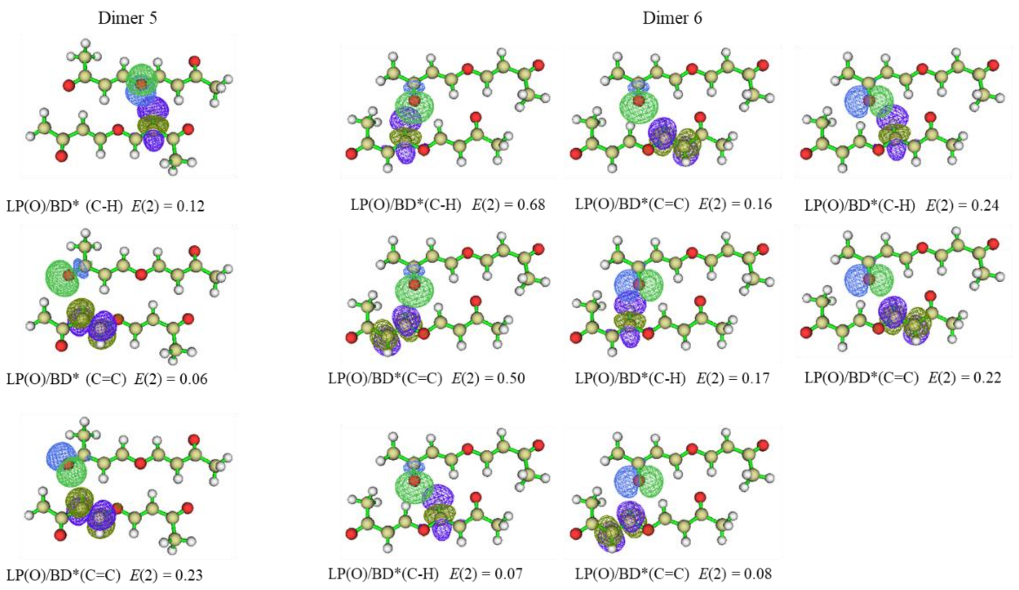

Supplementary Figure 39. Natural bond orbital (NBO) analysis with second-order perturbation of typical dimers of *EE*-OBBO. LP = lone pair, BD* = anti-bonding, *E*(2) = the stabilization energy with second-order perturbation (unit = kcal/mol).

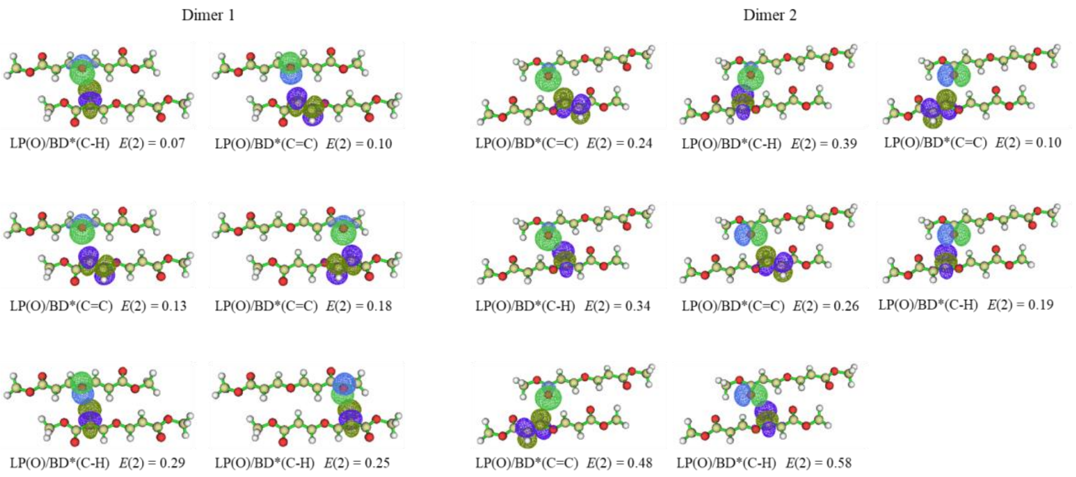

Supplementary Figure 40. Natural bond orbital (NBO) analysis with second-order perturbation of typical dimers of *EE*-DMODA. LP = lone pair, BD* = anti-bonding, *E*(2) = the stabilization energy with second-order perturbation (unit = kcal/mol).

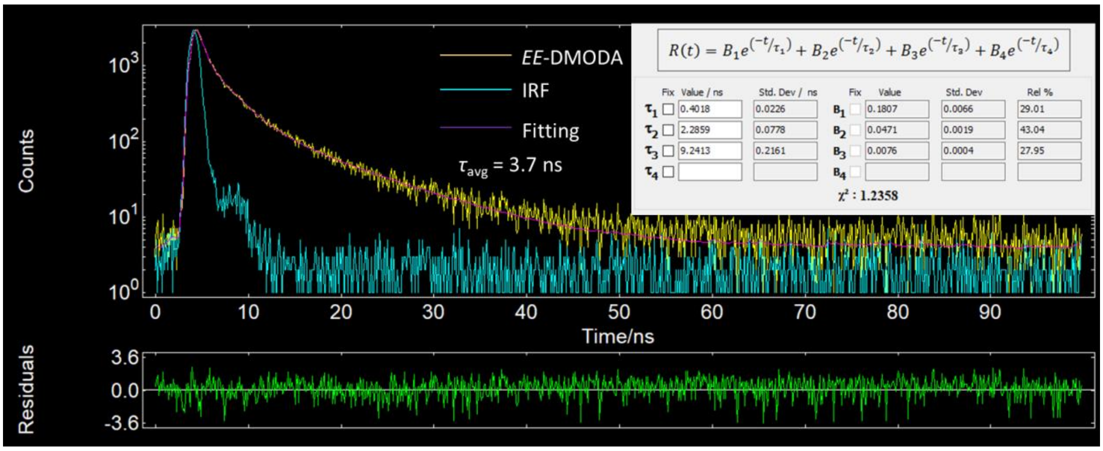

Supplementary Figure 41. Time-resolved photoluminescence decay curve of *EE*-DMODA with instrument response function (IRF) and time-correlated single photon counting (TCSPC) fitting parameters measured at the emission maximum of 445 nm. λ_{ex} = 365 nm.

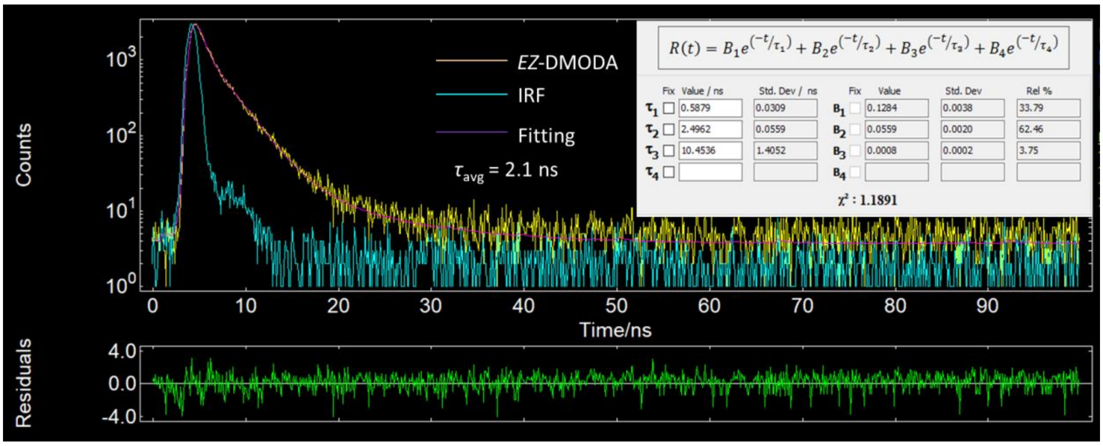

Supplementary Figure 42. Time-resolved photoluminescence decay curve of *EZ*-DMODA with IRF and TCSPC fitting parameters measured at the emission maximum of 478 nm. λ_{ex} = 365 nm.

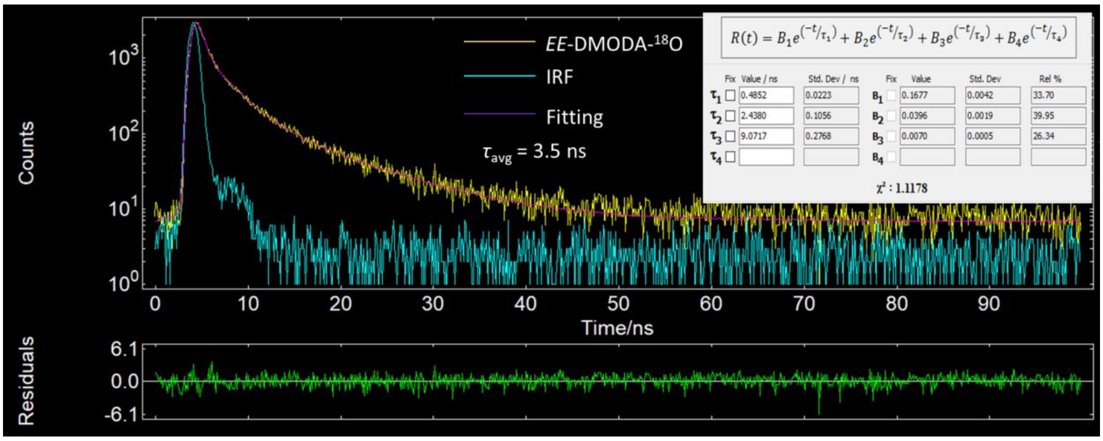

Supplementary Figure 43. Time-resolved photoluminescence decay curve of *EE*-DMODA-^{18}O with IRF and TCSPC fitting parameters measured at the emission maximum of 485 nm. λ_{ex} = 365 nm.

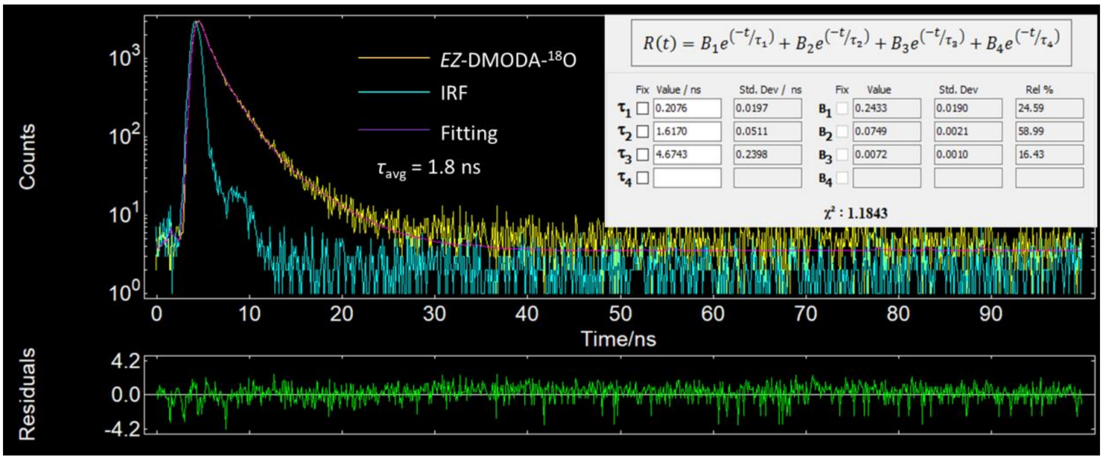

Supplementary Figure 44. Time-resolved photoluminescence decay curve of *EZ*-DMODA-^{18}O with IRF and TCSPC fitting parameters measured at the emission maximum of 493 nm. λ_{ex} = 365 nm.

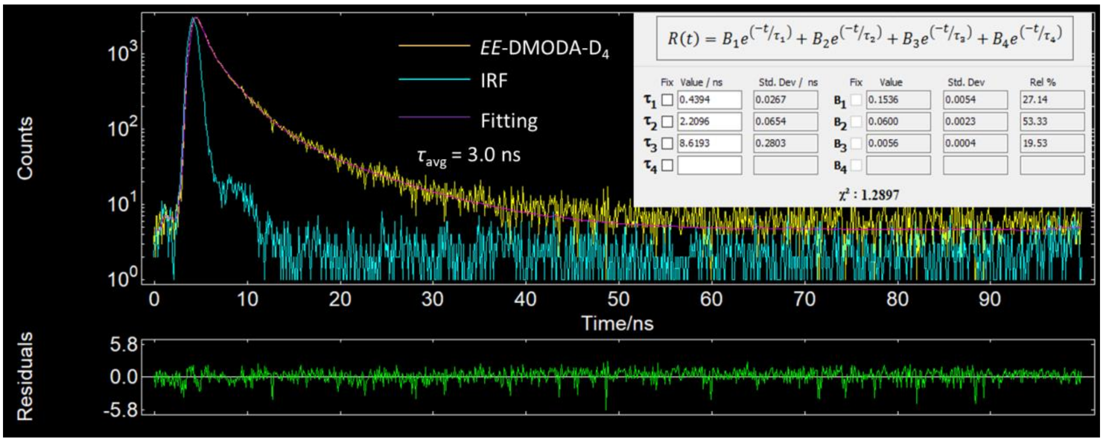

Supplementary Figure 45. Time-resolved photoluminescence decay curve of *EE*-DMODA-D_4 with IRF and TCSPC fitting parameters measured at the emission maximum of 437 nm. λ_{ex} = 365 nm.

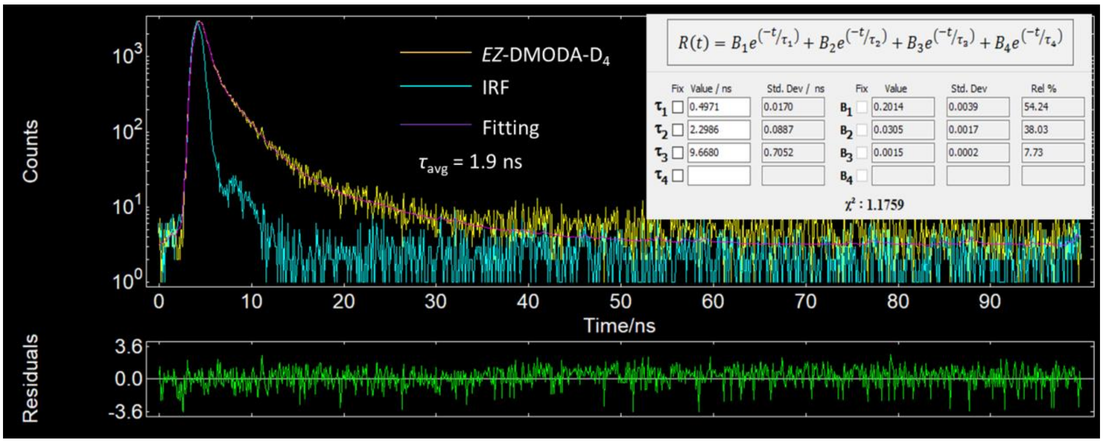

Supplementary Figure 46. Time-resolved photoluminescence decay curve of *EZ*-DMODA-D_4 with IRF and TCSPC fitting parameters measured at the emission maximum of 452 nm. λ_{ex} = 365 nm.

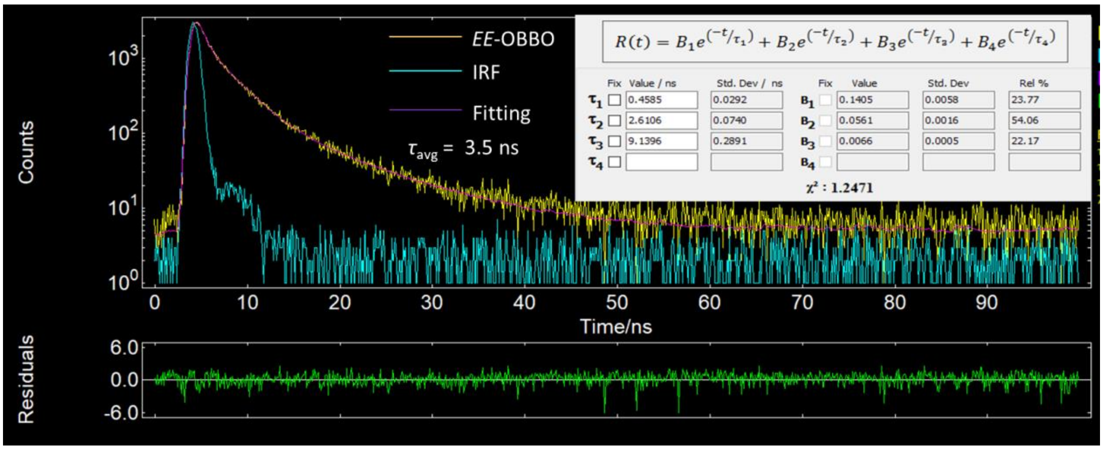

Supplementary Figure 47. Time-resolved photoluminescence decay curve of *EE*-OBBO with IRF and TCSPC fitting parameters measured at the emission maximum of 504 nm. λ_{ex} = 365 nm.

Synthetic procedure and characterization data for polymers

Procedures for the interfacial polymerization of H_2O and monomer TMP: 300 mg of monomer TMP was weighed and added into a 50 mL beaker. 15 mL of DCM was added to dissolve them completely, serving as the bottom layer in the beaker. Meanwhile, 200 mg of DABCO was dissolved in 15 mL of water to obtain the top layer. After reacting for 1 h at room temperature, a self-standing film could be obtained. Elemental analysis: Calcd for PTMP unit cell ($C_{30}H_{34}O_{15}$): C, 56.73%; H, 5.36%; Found: C, 56.40%; H, 6.25%.

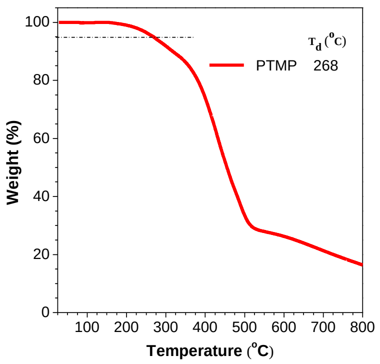

Supplementary Figure 48. TGA thermograms recorded under nitrogen at a heating rate of 10 K/min.

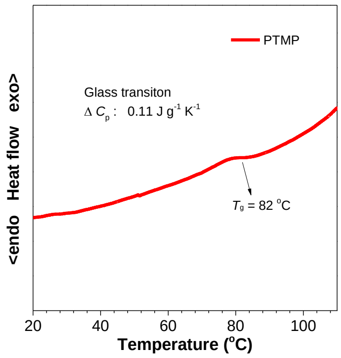

Supplementary Figure 49. DSC thermograms of PTMP recorded under a nitrogen atmosphere during the first heating cycle at a scan rate of 10 K/min. ($\Delta\ C_p$: specific heat capacity difference)

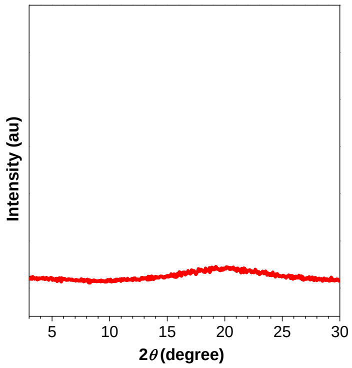

Supplementary Figure 50. PXRD patterns of PTMP.

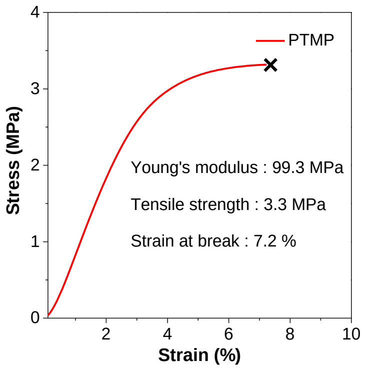

Supplementary Figure 51. Stress-strain curve for PTMP film (60 min) measured by tensile testing (1 N/min, room temperature, with the break point indicated by ×).

Photophysical properties of polymers

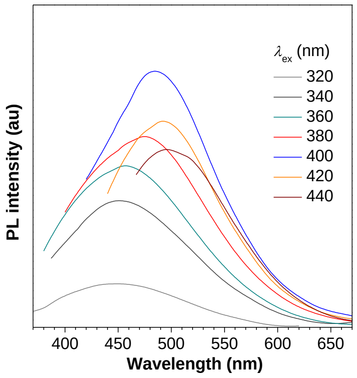

Supplementary Figure 52. Photoluminescence spectra of the polymer film at the polymerization time of 5 min in the solid state.

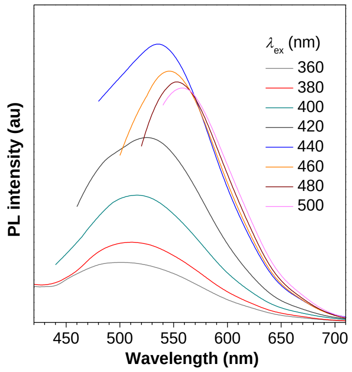

Supplementary Figure 53. Photoluminescence spectra of the polymer film at the polymerization time of 20 min in the solid state.

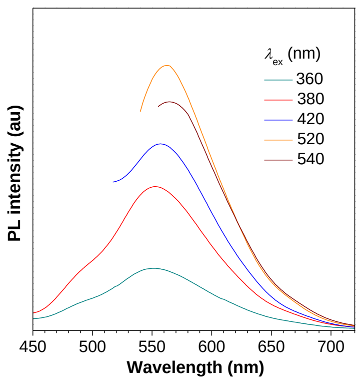

Supplementary Figure 54. Photoluminescence spectra of the polymer film at the polymerization time of 60 min in the solid state.

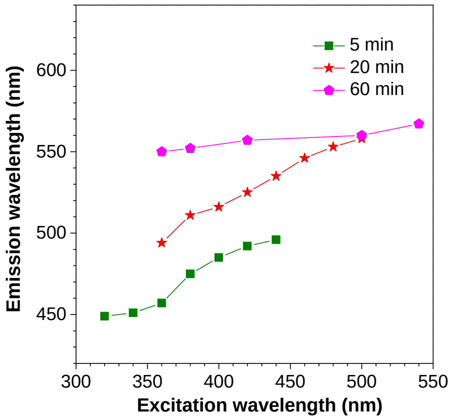

Supplementary Figure 55. The plots of emission wavelengths *versus* excitation wavelength.

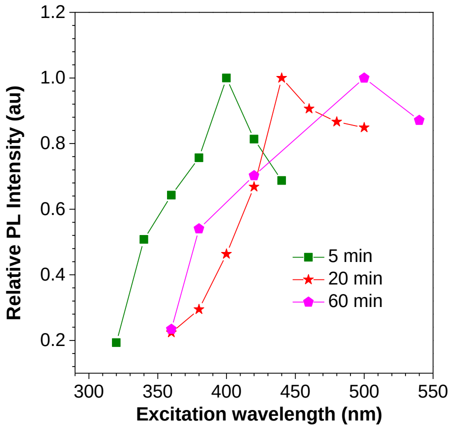

Supplementary Figure 56. Plots of relative photoluminescence intensities *versus* excitation wavelength.

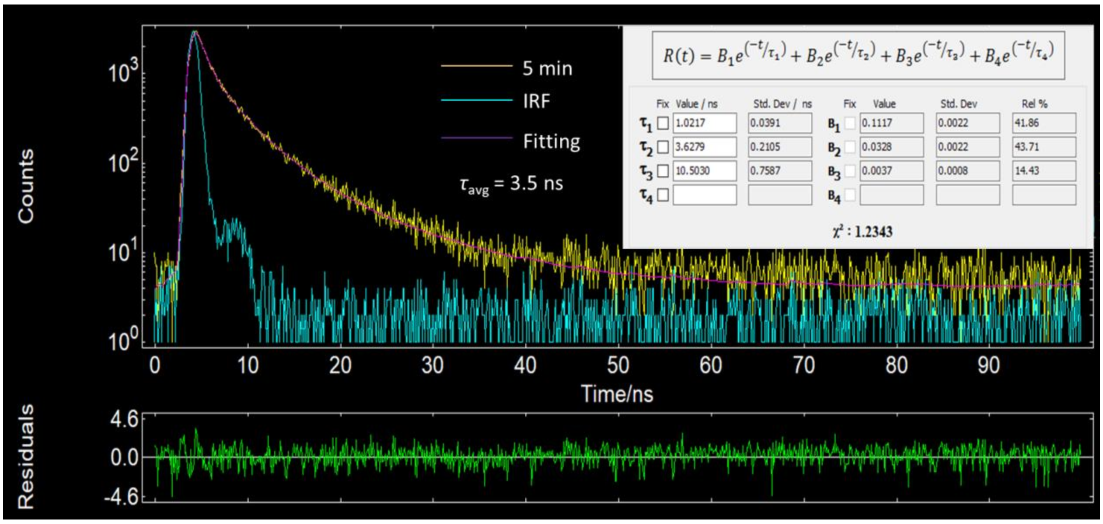

Supplementary Figure 57. Time-resolved photoluminescence decay curve of PTMP (5 min) with IRF and TCSPC fitting parameters measured at the emission maximum of 475 nm. λ_{ex} = 365 nm.

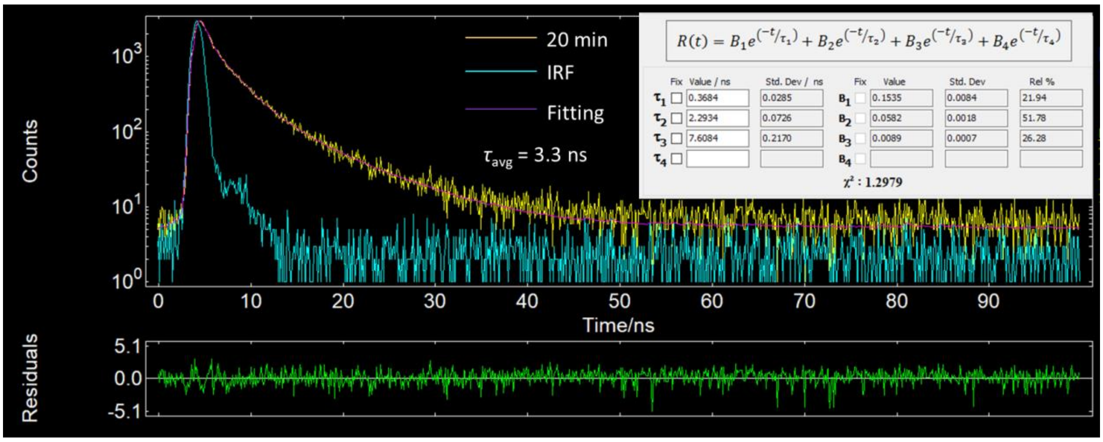

Supplementary Figure 58. Time-resolved photoluminescence decay curve of PTMP (20 min) with IRF and TCSPC fitting parameters measured at the emission maximum of 511 nm. λ_{ex} = 365 nm.

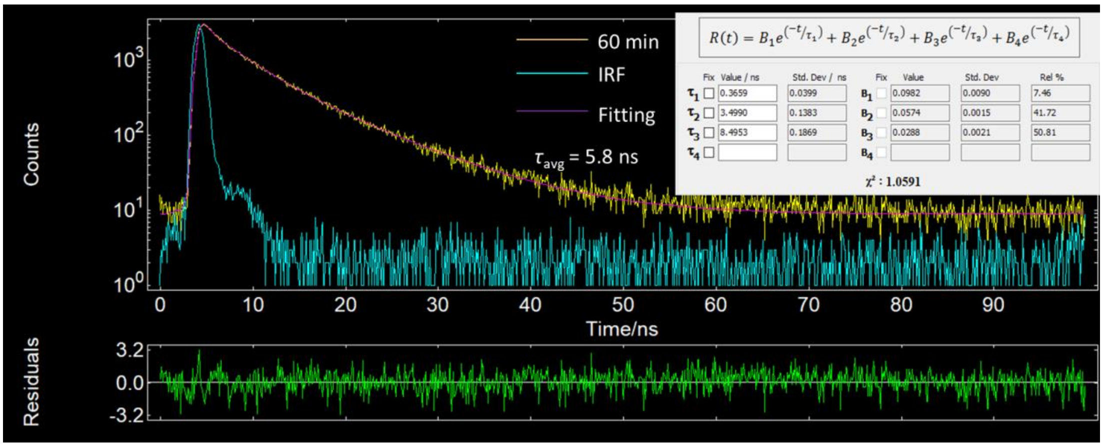

Supplementary Figure 59. Time-resolved photoluminescence decay curve of PTMP (60 min) with IRF and TCSPC fitting parameters measured at the emission maximum of 552 nm. λ_{ex} = 365 nm.

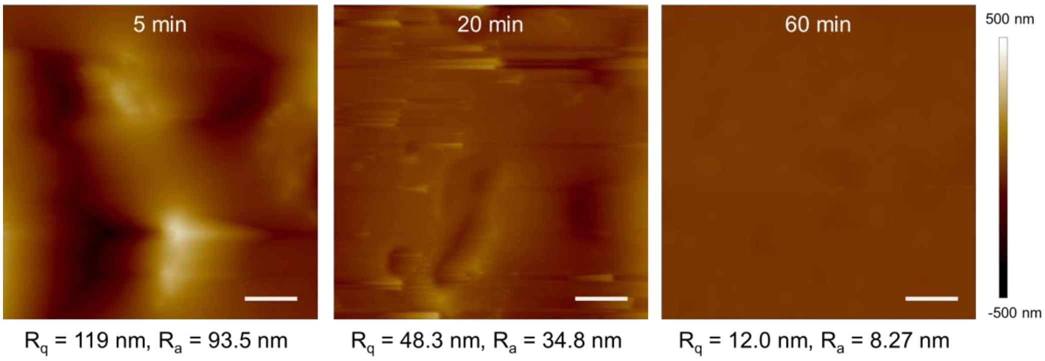

Supplementary Figure 60. Atomic force microscope of PTMP at different polymerization times on the water side (scale bar: 3 μm).

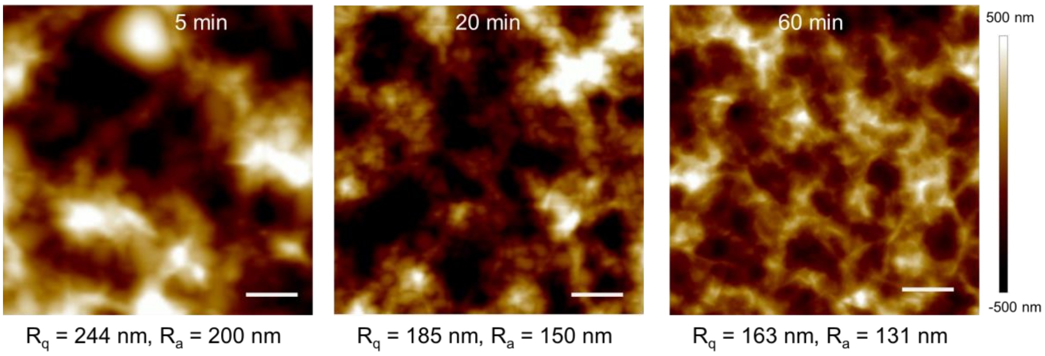

Supplementary Figure 61. Atomic force microscope of PTMP at different polymerization times on the DCM side (scale bar: 3 μm).

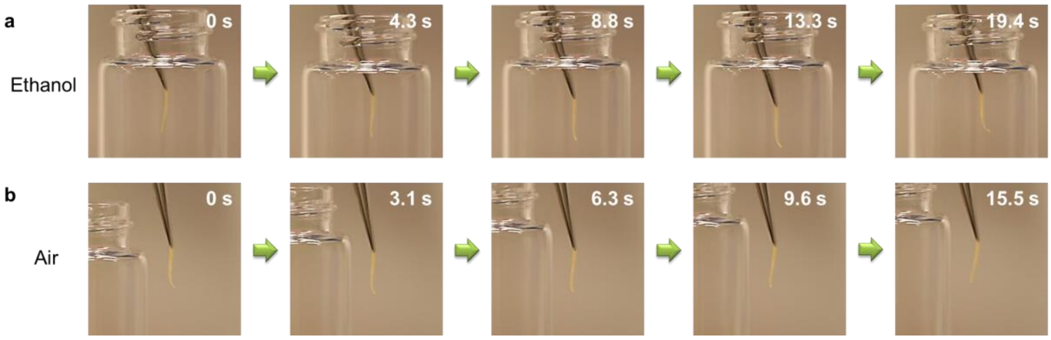

Supplementary Figure 62. Bending-recovering behavior of PTMP placed in (a) an ethanol vapor or (b) air atmosphere.

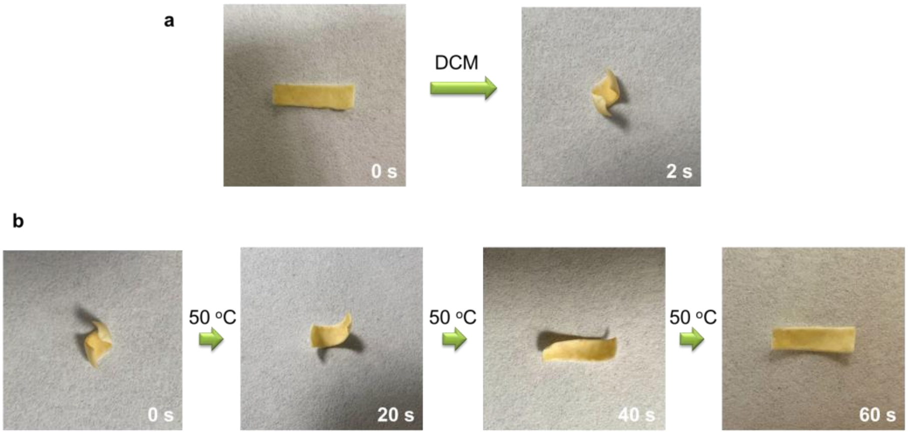

Supplementary Figure 63. Bending-recovering behavior of PTMP placed in (a) a DCM vapor or (b) at 50 ^{o}C.

Additional data

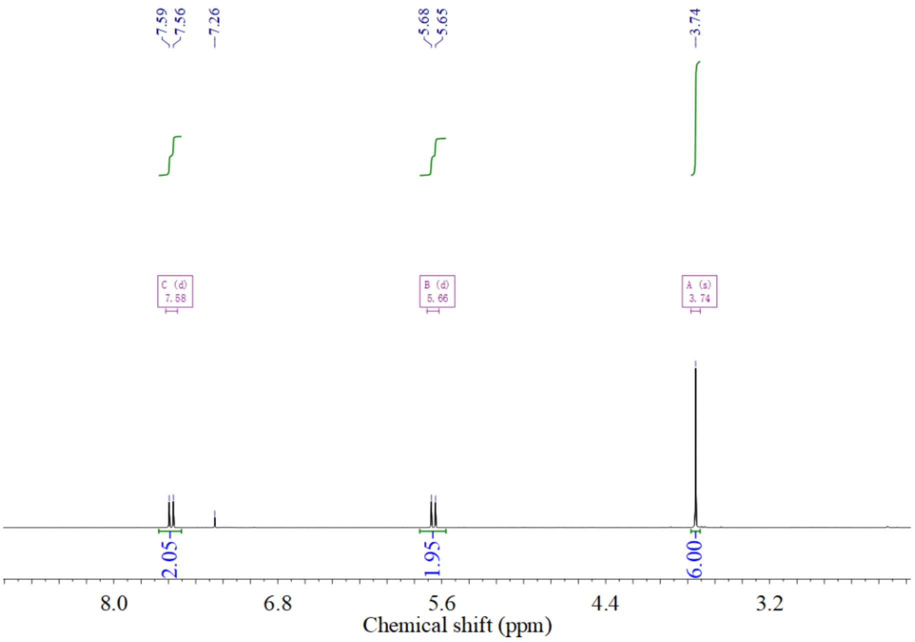

Supplementary Figure 64. ^{1}H NMR spectrum of *EE*-DMODA in $CDCl_3$.

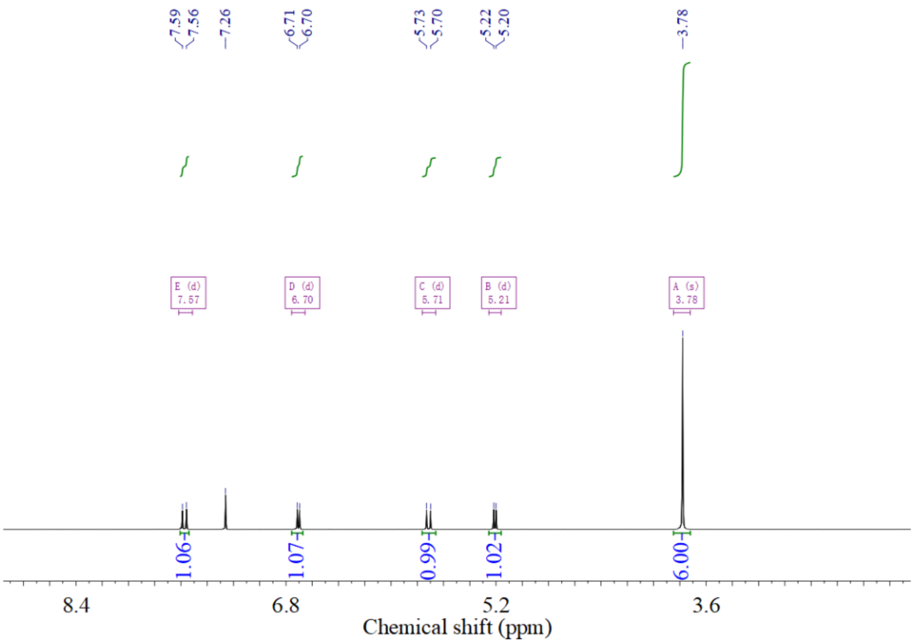

Supplementary Figure 65. ^{1}H NMR spectrum of *EZ*-DMODA in $CDCl_3$.

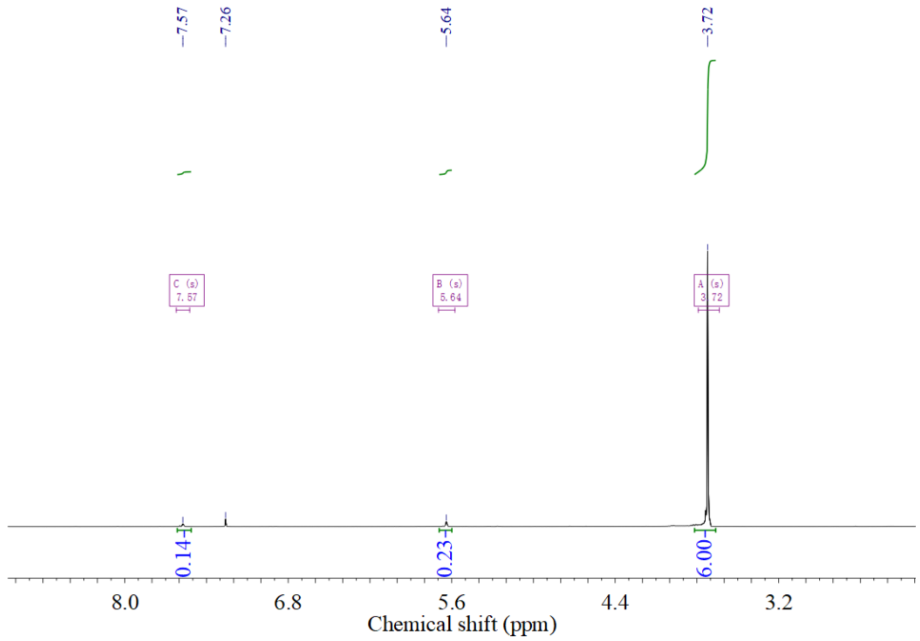

Supplementary Figure 66. ^{1}H NMR spectrum of *EE*-DMODA-D_4 in $CDCl_3$.

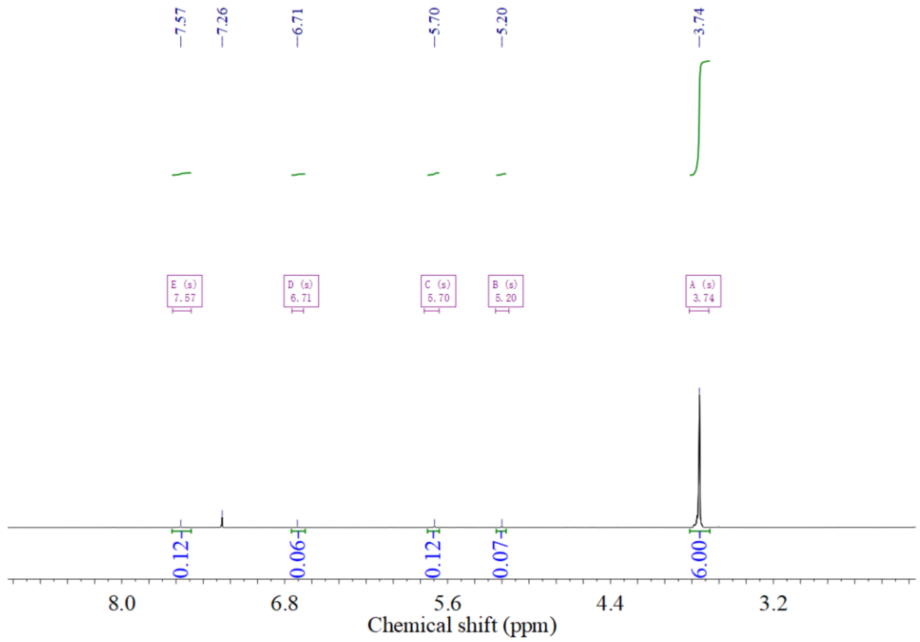

Supplementary Figure 67. ^{1}H NMR spectrum of *EZ*-DMODA-D_4 in $CDCl_3$.

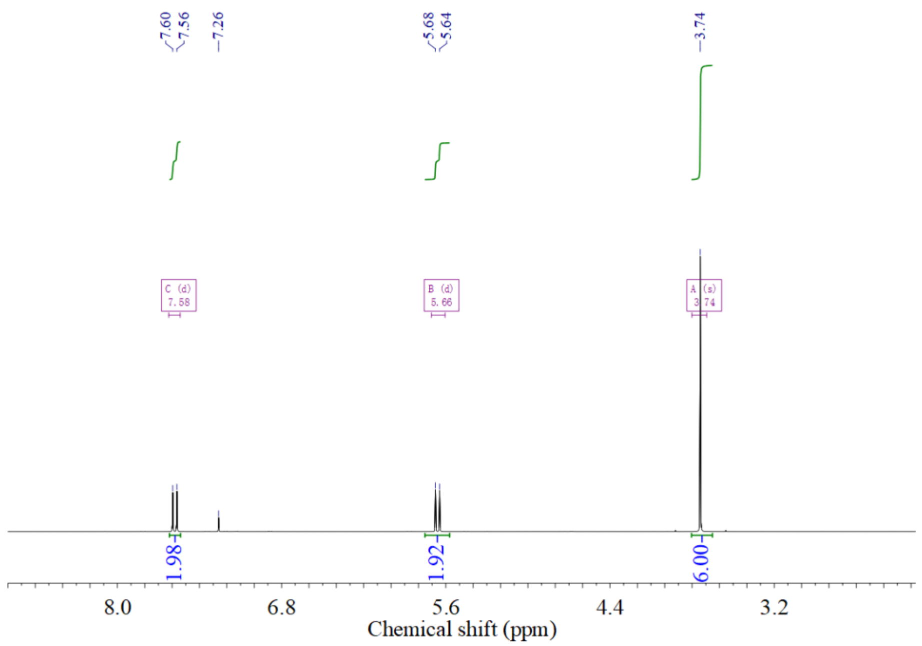

Supplementary Figure 68. ^{1}H NMR spectrum of *EE*-DMODA-^{18}O in $CDCl_3$.

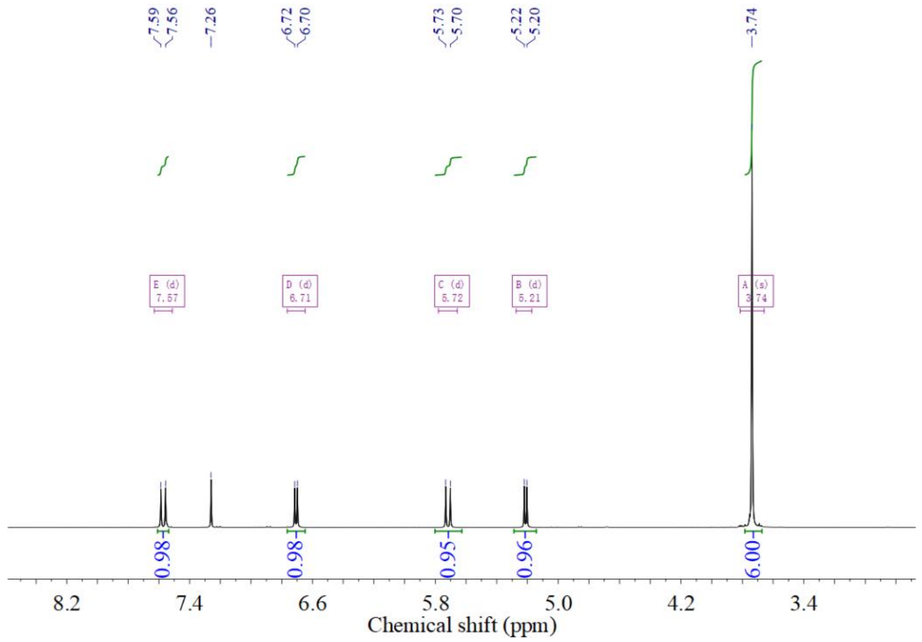

Supplementary Figure 69. ^{1}H NMR spectrum of *EZ*-DMODA-^{18}O in $CDCl_3$.

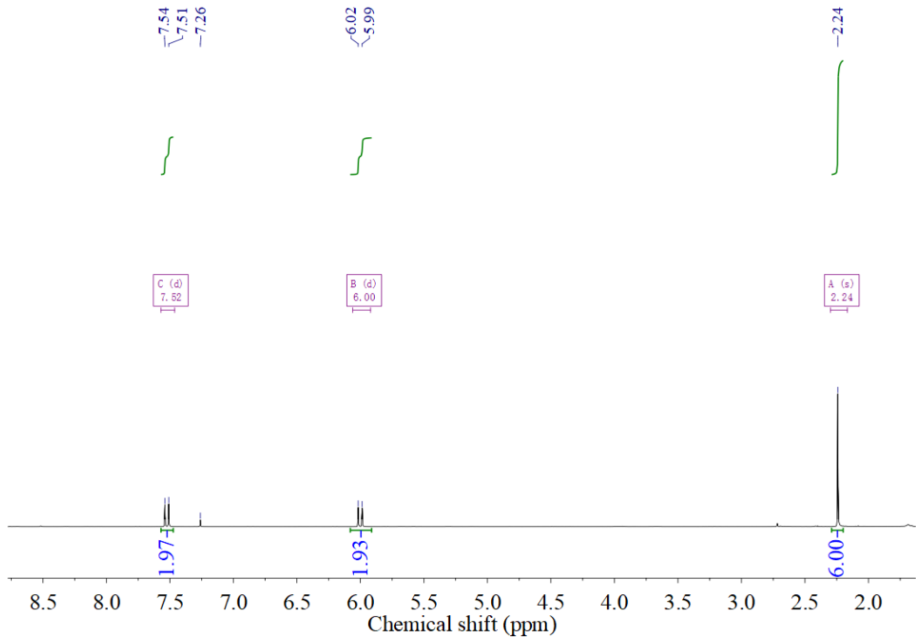

Supplementary Figure 70. ^{1}H NMR spectrum of *EE*-OBBO in $CDCl_3$.

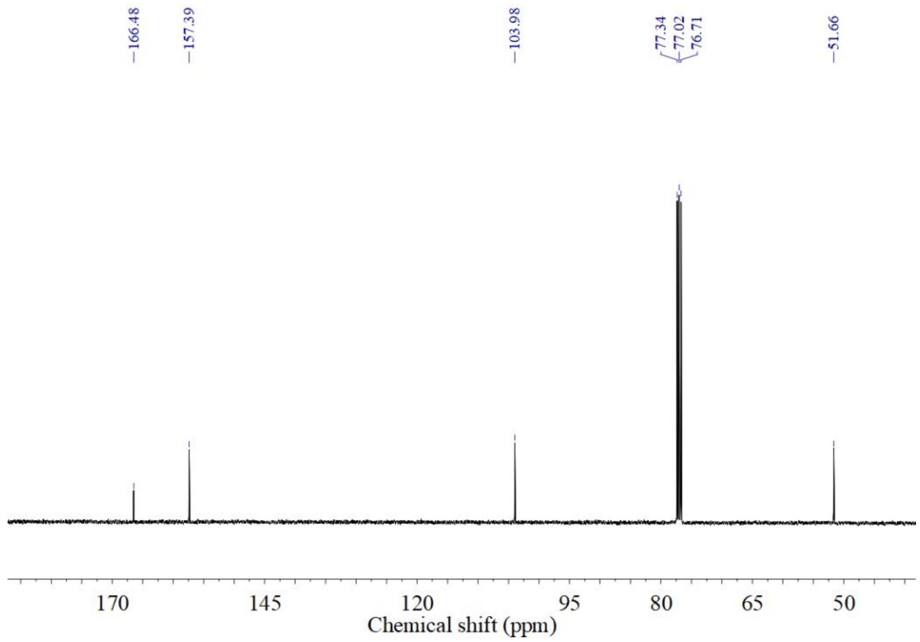

Supplementary Figure 71. ^{13}C NMR spectrum of *EE*-DMODA in $CDCl_3$.

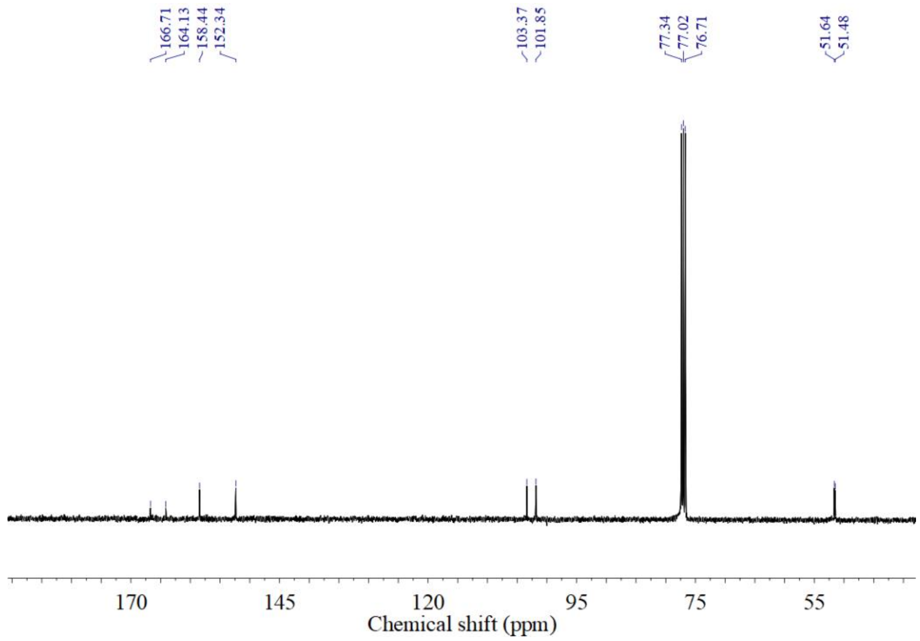

Supplementary Figure 72. ^{13}C NMR spectrum of *EZ*-DMODA in $CDCl_3$.

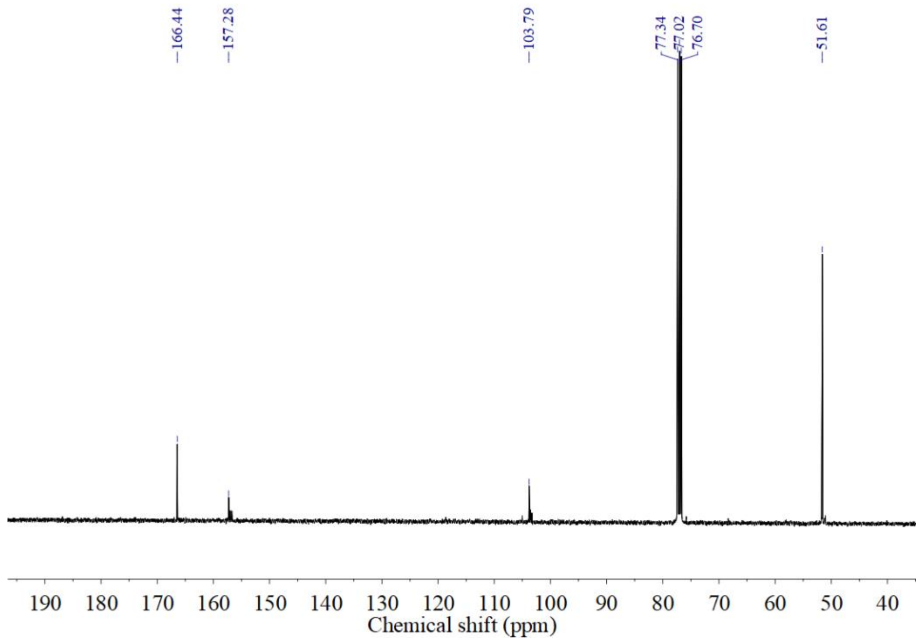

Supplementary Figure 73. ^{13}C NMR spectrum of *EE*-DMODA-D_4 in $CDCl_3$.

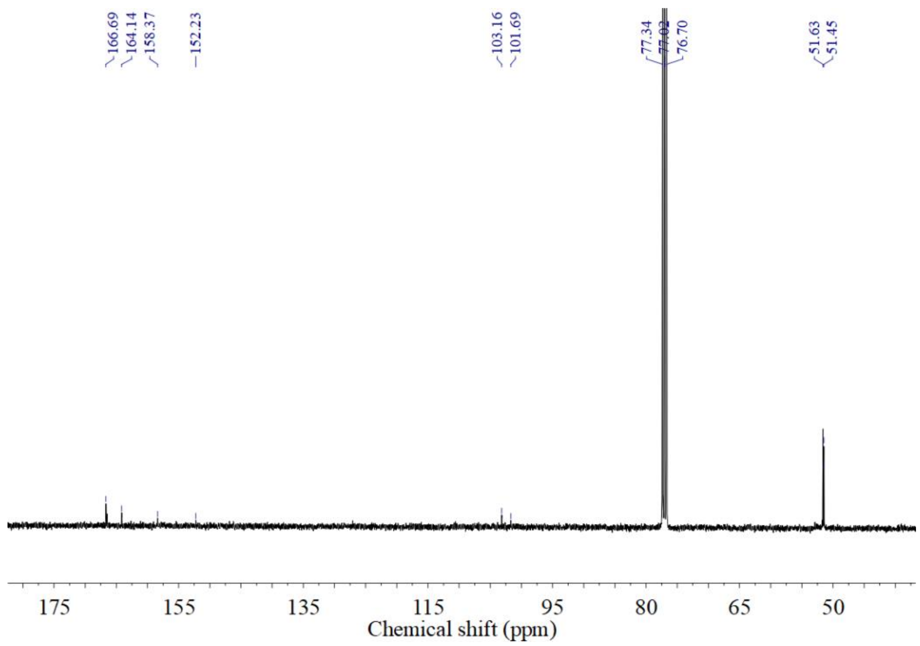

Supplementary Figure 74. ^{13}C NMR spectrum of *EZ*-DMODA-D_4 in $CDCl_3$.

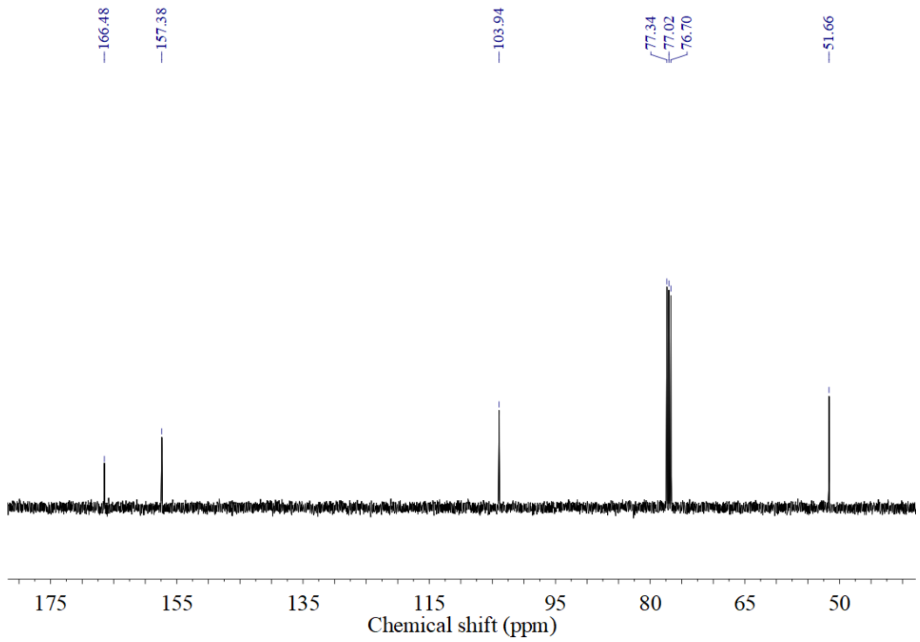

Supplementary Figure 75. ^{13}C NMR spectrum of *EE*-DMODA-^{18}O in $CDCl_3$.

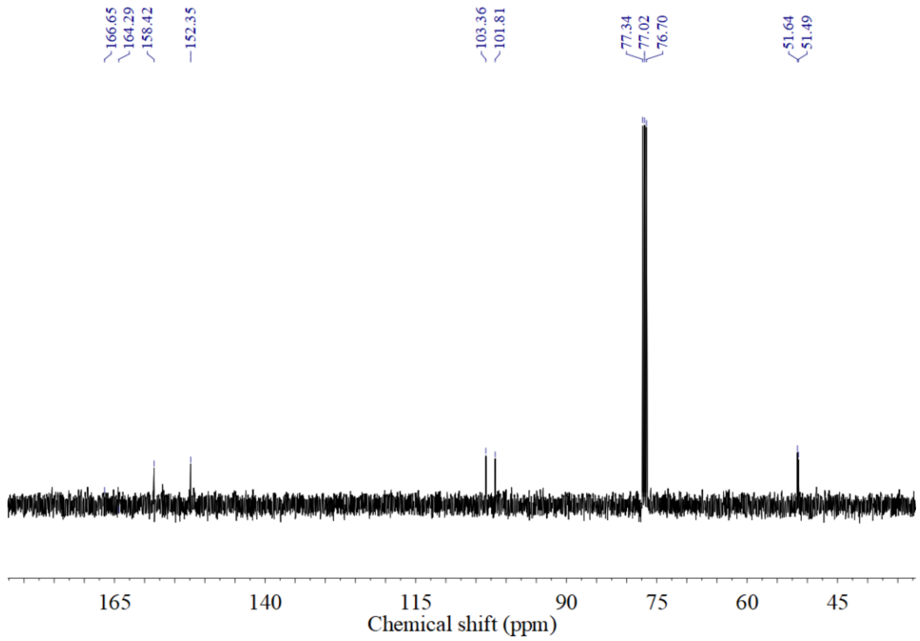

Supplementary Figure 76. ^{13}C NMR spectrum of *EZ*-DMODA-^{18}O in $CDCl_3$.

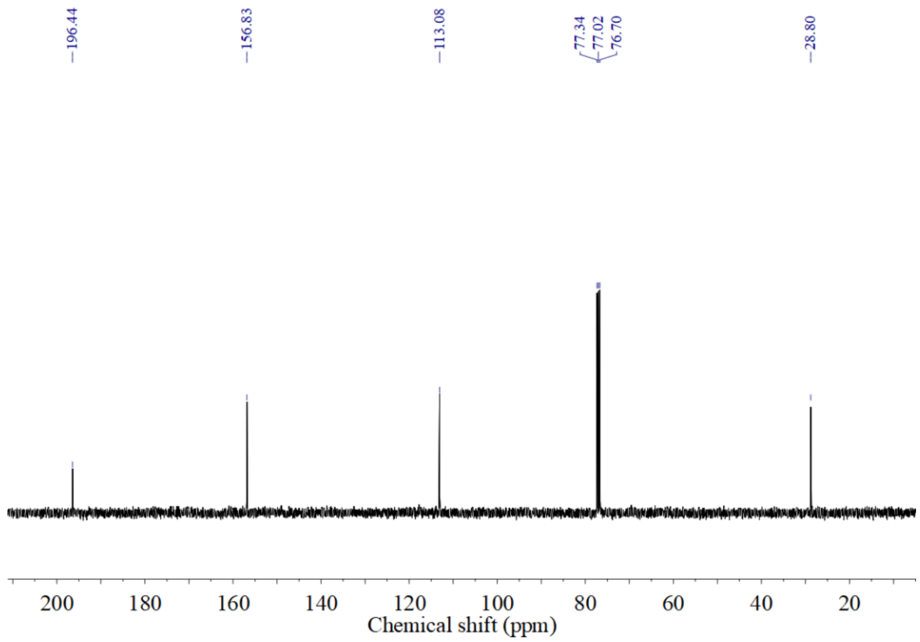

Supplementary Figure 77. ^{13}C NMR spectrum of *EE*-OBBO in $CDCl_3$.

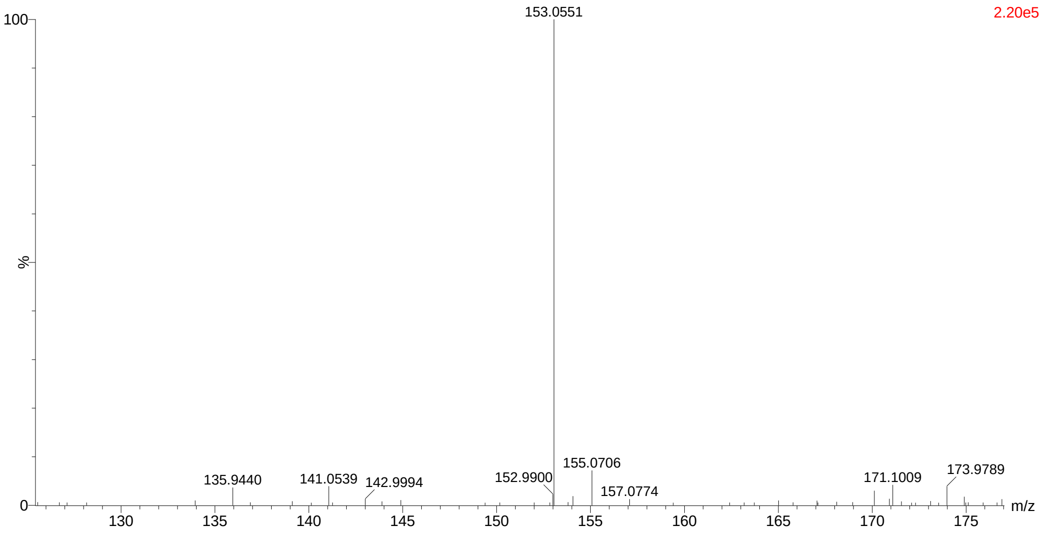

Supplementary Figure 78. HRMS of *EE*-OBBO**.**

Cartesian coordinates of optimized molecular geometry

Supplementary Table 3. Cartesian coordinates of optimized *EE*-DMODA in the excited state calculated at B3LYP-D3/6-31G(d,p) level.

	X	Y	Z
O	0.015543	-0.543209	-0.000064
C	2.364825	-0.609236	0.000122
H	2.381709	-1.691944	0.000211
O	4.688778	-0.715145	0.000126
C	1.207201	0.080984	-0.000026
H	1.200230	1.169751	-0.000116
O	-4.699676	-0.712485	0.000397
C	3.623390	0.133209	0.000159
C	-2.345160	-0.549655	-0.000171
H	-2.330881	-1.631863	0.000144
C	-1.144086	0.170672	-0.000248
H	-1.057590	1.250698	-0.000423
O	-3.836281	1.339376	-0.000198
C	-3.583605	0.040559	-0.000373
O	3.744998	1.349006	-0.000086
C	5.969048	-0.070777	-0.000021
H	6.705931	-0.874605	0.000043
H	6.089099	0.558272	0.886789
H	6.089012	0.558042	-0.887008
C	-5.994889	-0.073113	0.000262
H	-6.128085	0.539804	-0.894855
H	-6.127770	0.540837	0.894718

H	-6.708898	-0.895188	0.000866

Supplementary Table 4. Cartesian coordinates of optimized *EZ*-DMODA in the excited state calculated at B3LYP-D3/6-31G(d,p) level.

	X	Y	Z
O	3.790317	-0.569243	0.000057
O	-0.283002	-0.363904	-0.000124
O	-2.626855	1.018732	0.000053
O	3.963597	1.693200	-0.000156
O	-4.480709	-0.211027	0.000170
C	1.062668	-0.421929	-0.000076
H	1.504947	-1.415586	0.000029
C	3.265941	0.694309	-0.000120
C	1.805269	0.702884	-0.000162
H	1.334706	1.678156	-0.000265
C	-3.132330	-0.197304	0.000005
C	-1.041704	-1.497922	-0.000054
H	-0.517526	-2.445946	-0.000054
C	5.222244	-0.623460	0.000153
H	5.479090	-1.683445	0.000328
H	5.631350	-0.131339	0.887132
H	5.631458	-0.131608	-0.886925
C	-2.435246	-1.384600	-0.000022
H	-3.038250	-2.282387	0.000008
C	-5.202736	1.039488	0.000157
H	-6.253394	0.753266	0.000265
H	-4.971976	1.623955	-0.894248
H	-4.971823	1.624064	0.894450

Supplementary Table 5. Cartesian coordinates of optimized *EE*-OBBO in the excited state calculated at B3LYP-D3/6-31G(d,p) level.

	X	Y	Z
O	-0.005429	-0.617710	0.000041
O	-4.749717	-0.794934	0.000090
O	3.591506	1.438553	0.000097
C	-3.627977	-0.140715	-0.000177
C	-1.189329	0.054089	-0.000059
H	-1.134161	1.137210	-0.000180
C	-2.361891	-0.695329	0.000008
H	-2.276279	-1.775926	0.000254

C	3.590360	0.209555	0.000151
C	1.161646	0.054445	0.000065
H	1.111426	1.142323	0.000028
C	4.889336	-0.580026	0.000224
H	4.942816	-1.230263	-0.881000
H	4.942873	-1.229955	0.881673
H	5.735123	0.108381	0.000086
C	2.349646	-0.581836	0.000129
H	2.391634	-1.666282	0.000163
C	-3.900285	1.366589	-0.000516
H	-4.971396	1.572127	-0.001127
H	-3.450488	1.807109	-0.894822
H	-3.451461	1.807370	0.894154

Supplementary Table 6. Cartesian coordinates of optimized QM part of *EE*-DMODA (dimer 1) in the ground state calculated at B3LYP-D3/6-31G(d,p) level.

	X	Y	Z
O	-1.749340	1.199384	0.564835
C	0.554433	1.608386	0.494996
H	0.776645	0.646737	0.929575
O	2.851442	1.964040	0.435161
C	-0.699546	1.998286	0.246484
H	-0.913535	2.958396	-0.211428
O	-6.246056	0.067737	0.425114
C	1.655031	2.500643	0.108338
C	-4.023340	0.711610	0.458855
H	-3.861051	-0.179199	1.039972
C	-3.002343	1.511257	0.144417
H	-3.133133	2.409628	-0.448989
O	-5.692016	1.905021	-0.781778
C	-5.368723	0.994429	-0.043570
O	1.531729	3.573855	-0.449054
C	4.003563	2.739612	0.068850
H	4.841754	2.044468	0.075633
H	4.166057	3.545340	0.786451
H	3.867580	3.186571	-0.915392
C	-7.607503	0.284428	0.031632
H	-7.694341	0.360935	-1.052686
H	-7.988493	1.209035	0.471542
H	-8.165604	-0.571228	0.405876
O	1.749475	-1.199814	-0.564369

C	-0.554215	-1.608608	-0.494713
H	-0.776099	-0.646771	-0.929039
O	-2.851213	-1.963489	-0.435744
C	0.699675	-1.998798	-0.246265
H	0.913594	-2.959078	0.211322
O	6.245895	-0.067151	-0.424021
C	-1.655172	-2.500598	-0.108510
C	4.023254	-0.711461	-0.457966
H	3.860221	0.180286	-1.037466
C	3.002578	-1.511882	-0.144420
H	3.133650	-2.411018	0.447778
O	5.692480	-1.905792	0.781022
C	5.368816	-0.994467	0.043883
O	-1.532468	-3.573967	0.448743
C	-4.003827	-2.738615	-0.070144
H	-4.841825	-2.043295	-0.078703
H	-4.165527	-3.545112	-0.787067
H	-3.869154	-3.184584	0.914718
C	7.607523	-0.284347	-0.031350
H	7.694866	-0.361678	1.052878
H	7.988130	-1.208726	-0.472083
H	8.165621	0.571457	-0.405262

Supplementary Table 7. Cartesian coordinates of optimized QM part of *EE*-DMODA (dimer 2) in the ground state calculated at B3LYP-D3/6-31G(d,p) level.

	X	Y	Z
O	2.313275	2.022419	0.247359
C	0.047445	2.573549	0.162072
H	0.238656	3.540875	0.599383
O	-2.166142	3.232862	0.052848
C	1.036016	1.701927	-0.067987
H	0.860963	0.716563	-0.480182
O	6.847742	1.031556	0.249234
C	-1.323931	2.220859	-0.203960
C	4.575876	1.457071	0.247850
H	4.829759	2.361269	0.776864
C	3.312191	1.164059	-0.072439
H	3.038751	0.243180	-0.569094
O	5.520089	-0.521561	-0.726031
C	5.646841	0.540280	-0.136037
O	-1.683832	1.152210	-0.682057

C	-3.552581	3.006310	-0.259552
H	-4.066086	3.901029	0.087922
H	-3.944753	2.119438	0.238333
H	-3.683623	2.880453	-1.334495
C	7.970213	0.206977	-0.080091
H	8.001612	0.000745	-1.151614
H	7.918123	-0.747317	0.449004
H	8.850399	0.766105	0.232723
O	-2.313229	-2.022630	-0.248635
C	-0.047369	-2.573727	-0.162843
H	-0.238336	-3.541213	-0.599909
O	2.166379	-3.232686	-0.051676
C	-1.036028	-1.702111	0.066840
H	-0.861077	-0.716665	0.478917
O	-6.847596	-1.031429	-0.249812
C	1.323745	-2.220714	0.203731
C	-4.575880	-1.457426	-0.248298
H	-4.829935	-2.361367	-0.777691
C	-3.312129	-1.164516	0.071785
H	-3.038616	-0.243871	0.568830
O	-5.519773	0.520668	0.726971
C	-5.646676	-0.540671	0.136096
O	1.683178	-1.151726	0.681459
C	3.552297	-3.005100	0.262387
H	4.065667	-3.903095	-0.076656
H	3.946487	-2.122671	-0.241743
H	3.680817	-2.870302	1.336526
C	-7.970028	-0.206954	0.079835
H	-8.001066	-0.000582	1.151327
H	-7.918230	0.747300	-0.449410
H	-8.850255	-0.766206	-0.232639

Supplementary Table 8. Cartesian coordinates of optimized QM part of *EE*-OBBO (dimer 5) in the ground state calculated at B3LYP-D3/6-31G(d,p) level.

	X	Y	Z
O	1.004722	-1.319538	-0.607639
O	-3.609437	-2.090769	-0.487735
O	4.839571	-2.298186	0.676533
C	-2.494248	-2.517438	-0.204298
C	-0.047135	-2.110471	-0.290797
H	0.197216	-3.059767	0.170146

C	-1.304402	-1.728619	-0.552239
H	-1.485228	-0.767413	-1.012520
C	4.659603	-1.280256	0.023448
C	2.263092	-1.688342	-0.222303
H	2.370288	-2.640899	0.290346
C	5.786367	-0.336650	-0.331711
H	5.566114	0.659265	0.067177
H	5.867614	-0.230996	-1.418641
H	6.724091	-0.718538	0.072231
C	3.309708	-0.891153	-0.451297
H	3.180174	0.065809	-0.932299
C	-2.345159	-3.854283	0.506054
H	-3.299648	-4.111906	0.960542
H	-1.568924	-3.844439	1.275274
H	-2.096443	-4.633787	-0.218951
O	-1.004746	1.319483	0.607492
O	3.609413	2.090627	0.487906
O	-4.839553	2.298240	-0.676671
C	2.494261	2.517399	0.204482
C	0.047129	2.110420	0.290706
H	-0.197214	3.059774	-0.170125
C	1.304379	1.728514	0.552133
H	1.485164	0.767231	1.012277
C	-4.659611	1.280300	-0.023588
C	-2.263096	1.688304	0.222147
H	-2.370288	2.640866	-0.290496
C	-5.786400	0.336705	0.331525
H	-5.566149	-0.659206	-0.067367
H	-5.867662	0.231055	1.418449
H	-6.724119	0.718615	-0.072426
C	-3.309737	0.891144	0.451137
H	-3.180241	-0.065812	0.932181
C	2.345263	3.854488	-0.505438
H	3.299737	4.112157	-0.959933
H	1.568949	3.845014	-1.274594
H	2.096703	4.633765	0.219874

Supplementary Table 9. Cartesian coordinates of optimized QM part of *EE*-OBBO (dimer 6) in the ground state calculated at B3LYP-D3/6-31G(d,p) level.

	X	Y	Z
O	-2.256494	1.992158	-0.192898

O	-6.741727	0.622689	-0.296641
O	1.696917	1.204330	0.830229
C	-5.599243	0.372322	0.057601
C	-3.235533	1.079563	0.078973
H	-2.888857	0.137265	0.473461
C	-4.515833	1.340899	-0.195852
H	-4.798119	2.280920	-0.648448
C	1.377352	2.285462	0.332747
C	-0.984411	1.699810	0.142258
H	-0.799966	0.735072	0.603935
C	2.418168	3.366901	0.109842
H	2.057431	4.354100	0.406542
H	2.668750	3.426759	-0.954666
H	3.319422	3.124178	0.672180
C	0.006638	2.578925	-0.087647
H	-0.214634	3.524809	-0.559062
C	-5.259856	-0.941601	0.747528
H	-6.177406	-1.508071	0.897051
H	-4.772742	-0.772748	1.712838
H	-4.566878	-1.521700	0.132140
O	2.255422	-1.990053	0.195050
O	6.742995	-0.629641	0.292629
O	-1.698517	-1.199187	-0.822011
C	5.600528	-0.376004	-0.059366
C	3.234510	-1.076869	-0.074984
H	2.887623	-0.132139	-0.464013
C	4.515194	-1.342303	0.194360
H	4.796630	-2.285449	0.641089
C	-1.377820	-2.283750	-0.332696
C	0.982888	-1.696287	-0.136978
H	0.797175	-0.727336	-0.589404
C	-2.418402	-3.366916	-0.117032
H	-2.050474	-4.356125	-0.397295
H	-2.686888	-3.415663	0.943702
H	-3.311468	-3.131725	-0.695628
C	-0.006683	-2.579234	0.084857
H	0.216353	-3.529397	0.546908
C	5.263407	0.938969	-0.748345
H	6.182314	1.502506	-0.900431
H	4.773198	0.771461	-1.712354
H	4.574352	1.521592	-0.131016

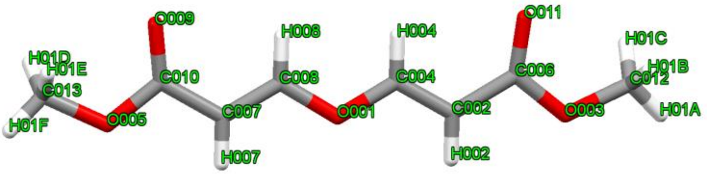

Supplementary Figure 79. The labels of *EE*-DMODA.

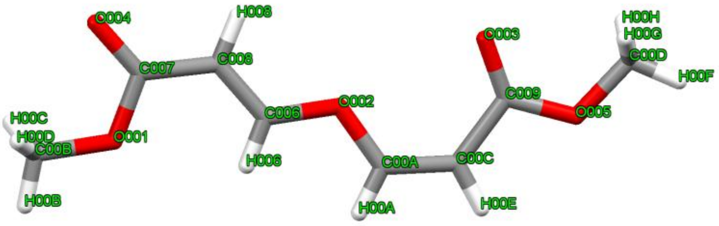

Supplementary Figure 80. The labels of *EZ*-DMODA.

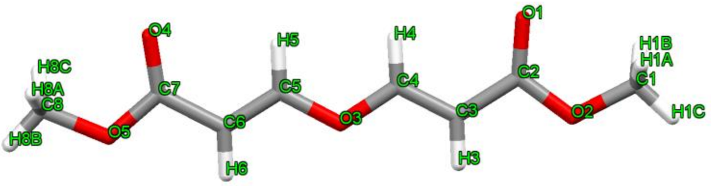

Supplementary Figure 81. The labels of *EE*-DMODA-^{18}O.

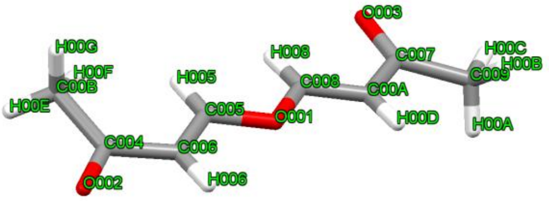

Supplementary Figure 82. The labels of *EE*-OBBO.

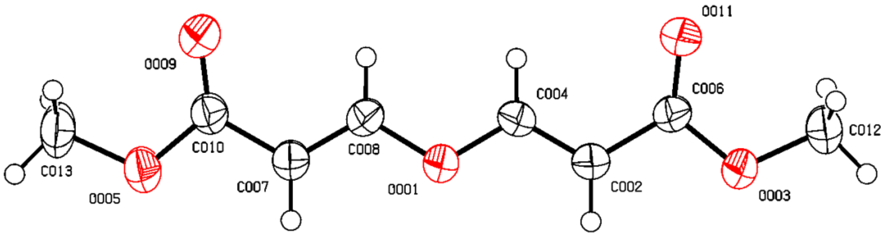

Supplementary Figure 83. ORTEP-style illustration of *EE*-DMODA with probability ellipsoids.

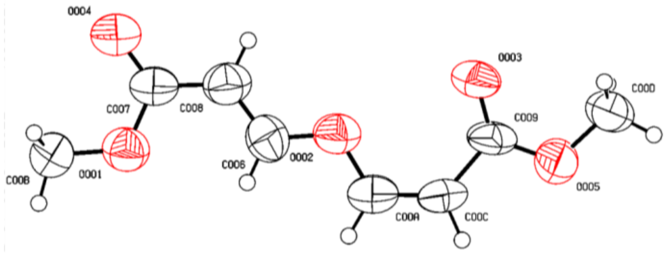

Supplementary Figure 84. ORTEP-style illustration of *EZ*-DMODA with probability ellipsoids.

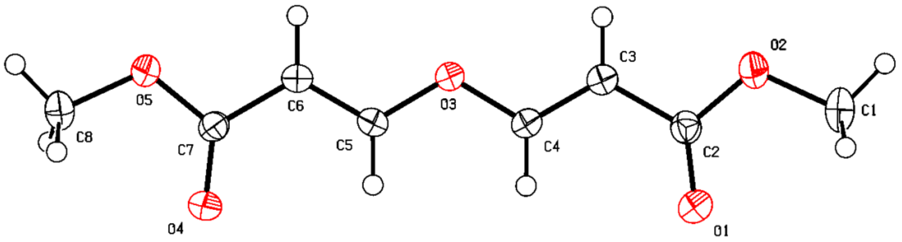

Supplementary Figure 85. ORTEP-style illustration of *EE*-DMODA-^{18}O with probability ellipsoids.

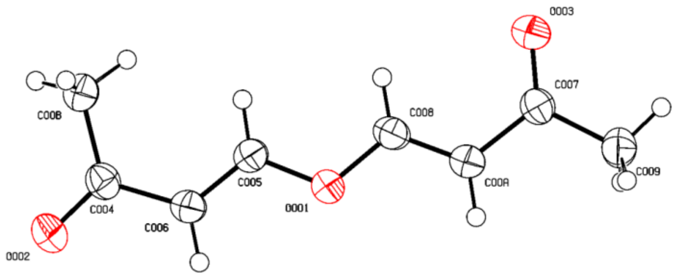

Supplementary Figure 86. ORTEP-style illustration of *EE*-OBBO with probability ellipsoids.

Alert level B

```
PLAT029_ALERT_3_B _diffrn_measured_fraction_theta_full value Low .      0.951 Why?
```

Supplementary Figure 87. CheckCif file B-level alert of *EE*-DMODA originated from the poor quality of collected data due to the crystal quality.

```
Alert level A
PLAT029_ALERT_3_A _diffrn_measured_fraction_theta_full value Low .      0.821 Why?
PLAT084_ALERT_3_A High wR2 Value (i.e. > 0.25) ...................      0.46 Report

Alert level B
PLAT082_ALERT_2_B High R1 Value ..................................      0.19 Report
PLAT340_ALERT_3_B Low Bond Precision on  C-C Bonds ...............   0.01375 Ang.
```

Supplementary Figure 88. CheckCif file A- and B-level alerts of *EZ*-DMODA originated from the poor quality of collected data due to the crystal quality.

Supplementary Table 10. Bond Lengths for *EE*-DMODA.

Atom1	Atom2	Length/Å
O001	C004	1.364(3)
O001	C008	1.372(2)
C002	H002	0.93
C002	C004	1.326(3)
C002	C006	1.466(3)
O003	C006	1.349(2)
O003	C012	1.443(3)
C004	H004	0.93
O005	C010	1.354(3)
O005	C013	1.448(3)
C006	O011	1.198(3)
C007	H007	0.93
C007	C008	1.330(3)
C007	C010	1.455(3)
C008	H008	0.93
O009	C010	1.206(3)
C012	H01A	0.96
C012	H01B	0.96
C012	H01C	0.96
C013	H01D	0.96
C013	H01E	0.96
C013	H01F	0.96

Supplementary Table 11. Bond Angles for *EE*-DMODA.

Atom1	Atom2	Atom3	Angle/°
C004	O001	C008	118.1(2)
H002	C002	C004	120.2
H002	C002	C006	120.2
C004	C002	C006	119.6(2)
C006	O003	C012	116.1(2)

O001	C004	C002	119.4(2)
O001	C004	H004	120.3
C002	C004	H004	120.3
C010	O005	C013	115.1(2)
C002	C006	O003	110.4(2)
C002	C006	O011	126.6(2)
O003	C006	O011	123.0(2)
H007	C007	C008	119.9
H007	C007	C010	119.8
C008	C007	C010	120.3(2)
O001	C008	C007	118.8(2)
O001	C008	H008	120.6
C007	C008	H008	120.6
O005	C010	C007	110.3(2)
O005	C010	O009	122.8(2)
C007	C010	O009	126.8(2)
O003	C012	H01A	109.5
O003	C012	H01B	109.5
O003	C012	H01C	109.5
H01A	C012	H01B	109.5
H01A	C012	H01C	109.5
H01B	C012	H01C	109.4
O005	C013	H01D	109.5
O005	C013	H01E	109.5
O005	C013	H01F	109.5
H01D	C013	H01E	109.4
H01D	C013	H01F	109.5
H01E	C013	H01F	109.5

Supplementary Table 12. Torsion Angles for *EE*-DMODA.

Atom	Atom	Atom	Atom	Torsion angles/°
C008	O001	C004	C002	-178.1(2)
C008	O001	C004	H004	1.9
C004	O001	C008	C007	179.7(2)
C004	O001	C008	H008	-0.3
H002	C002	C004	O001	0.1
H002	C002	C004	H004	-179.9
C006	C002	C004	O001	-179.9(2)
C006	C002	C004	H004	0.1
H002	C002	C006	O003	3.3
H002	C002	C006	O011	-175.2
C004	C002	C006	O003	-176.6(2)
C004	C002	C006	O011	4.8(4)

C012	O003	C006	C002	179.2(2)
C012	O003	C006	O011	-2.2(3)
C006	O003	C012	H01A	-173.7
C006	O003	C012	H01B	66.3
C006	O003	C012	H01C	-53.7
C013	O005	C010	C007	179.1(2)
C013	O005	C010	O009	-0.7(3)
C010	O005	C013	H01D	68.9
C010	O005	C013	H01E	-51.1
C010	O005	C013	H01F	-171.1
H007	C007	C008	O001	0.1
H007	C007	C008	H008	-179.9
C010	C007	C008	O001	-179.9(2)
C010	C007	C008	H008	0.1
H007	C007	C010	O005	-4.9
H007	C007	C010	O009	174.8
C008	C007	C010	O005	175.1(2)
C008	C007	C010	O009	-5.2(4)

Supplementary Table 13. Bond Lengths for *EZ*-DMODA.

Atom1	Atom2	Length/Å
O001	C007	1.33(1)
O001	C00B	1.43(1)
O002	C006	1.34(1)
O002	C00A	1.35(1)
O003	C009	1.17(1)
O004	C007	1.23(1)
O005	C009	1.32(1)
O005	C00D	1.41(1)
C006	H006	0.93
C006	C008	1.31(1)
C007	C008	1.41(2)
C008	H008	0.93
C009	C00C	1.46(1)
C00A	H00A	0.929
C00A	C00C	1.34(1)
C00B	H00B	0.96
C00B	H00C	0.96
C00B	H00D	0.96
C00C	H00E	0.93
C00D	H00F	0.96
C00D	H00G	0.96
C00D	H00H	0.96

Supplementary Table 14. Bond Angles for *EZ*-DMODA.

Atom1	Atom2	Atom3	Angle/°
C007	O001	C00B	118.4(7)
C006	O002	C00A	116.9(7)
C009	O005	C00D	117.3(7)
O002	C006	H006	118.8
O002	C006	C008	122.5(8)
H006	C006	C008	118.7
O001	C007	O004	119.3(8)
O001	C007	C008	115.8(8)
O004	C007	C008	124.9(9)
C006	C008	C007	125(1)
C006	C008	H008	118
C007	C008	H008	118
O003	C009	O005	125.0(8)
O003	C009	C00C	124.0(8)
O005	C009	C00C	110.9(7)
O002	C00A	H00A	118.8
O002	C00A	C00C	122.4(8)
H00A	C00A	C00C	118.9
O001	C00B	H00B	109.5
O001	C00B	H00C	109.5
O001	C00B	H00D	109.5
H00B	C00B	H00C	109.5
H00B	C00B	H00D	109.4
H00C	C00B	H00D	109.4
C009	C00C	C00A	129.2(8)
C009	C00C	H00E	115.4
C00A	C00C	H00E	115.5
O005	C00D	H00F	109.4
O005	C00D	H00G	109.4
O005	C00D	H00H	109.5
H00F	C00D	H00G	109
H00F	C00D	H00H	110
H00G	C00D	H00H	110

Supplementary Table 15. Torsion Angles for *EZ*-DMODA.

Atom1	Atom2	Atom3	Atom4	Torsion angles/°
C00B	O001	C007	O004	1(1)
C00B	O001	C007	C008	179.9(8)
C007	O001	C00B	H00B	-179.1
C007	O001	C00B	H00C	61
C007	O001	C00B	H00D	-59

C00A	O002	C006	H006	-3
C00A	O002	C006	C008	177.5(9)
C006	O002	C00A	H00A	3
C006	O002	C00A	C00C	-176.8(8)
C00D	O005	C009	O003	-2(1)
C00D	O005	C009	C00C	-178.2(7)
C009	O005	C00D	H00F	175.1
C009	O005	C00D	H00G	55
C009	O005	C00D	H00H	-65
O002	C006	C008	C007	179.6(9)
O002	C006	C008	H008	0
H006	C006	C008	C007	0
H006	C006	C008	H008	180
O001	C007	C008	C006	-1(2)
O001	C007	C008	H008	179.1
O004	C007	C008	C006	178(1)
O004	C007	C008	H008	-2
O003	C009	C00C	C00A	8(2)
O003	C009	C00C	H00E	-172.2
O005	C009	C00C	C00A	-175.6(9)
O005	C009	C00C	H00E	4
O002	C00A	C00C	C009	-1(1)
O002	C00A	C00C	H00E	178.8
H00A	C00A	C00C	C009	178.5
H00A	C00A	C00C	H00E	-1

Supplementary Table 16. Bond Lengths for *EE*-DMODA-^{18}O.

Atom1	Atom2	Length/Å
O1	C2	1.207(2)
O2	C1	1.443(2)
O2	C2	1.351(2)
O3	C4	1.368(2)
O3	C5	1.363(2)
O4	C7	1.208(2)
O5	C7	1.343(2)
O5	C8	1.441(2)
C1	H1A	0.98
C1	H1B	0.98
C1	H1C	0.98
C2	C3	1.464(2)
C3	H3	0.95
C3	C4	1.326(2)
C4	H4	0.95
C5	H5	0.95

C5	C6	1.327(2)
C6	H6	0.95
C6	C7	1.462(2)
C8	H8A	0.98
C8	H8B	0.98
C8	H8C	0.98

Supplementary Table 17. Bond Angles for *EE*-DMODA-^{18}O.

Atom1	Atom2	Atom3	Angle/°
C1	O2	C2	115.2(1)
C4	O3	C5	118.3(1)
C7	O5	C8	116.1(1)
O2	C1	H1A	109.5
O2	C1	H1B	109.5
O2	C1	H1C	109.4
H1A	C1	H1B	109.5
H1A	C1	H1C	109.5
H1B	C1	H1C	109.5
O1	C2	O2	123.3(1)
O1	C2	C3	126.4(1)
O2	C2	C3	110.3(1)
C2	C3	H3	119.8
C2	C3	C4	120.4(1)
H3	C3	C4	119.8
O3	C4	C3	118.9(1)
O3	C4	H4	120.5
C3	C4	H4	120.5
O3	C5	H5	120.3
O3	C5	C6	119.4(1)
H5	C5	C6	120.3
C5	C6	H6	120
C5	C6	C7	119.9(1)
H6	C6	C7	120.1
O4	C7	O5	122.9(1)
O4	C7	C6	126.3(1)
O5	C7	C6	110.8(1)
O5	C8	H8A	109.5
O5	C8	H8B	109.5
O5	C8	H8C	109.5
H8A	C8	H8B	109.5
H8A	C8	H8C	109.5
H8B	C8	H8C	109.5

Supplementary Table 18. Torsion Angles for *EE*-DMODA-^{18}O.

Atom1	Atom2	Atom3	Atom4	Torsion angles/°
C2	O2	C1	H1A	57.9
C2	O2	C1	H1B	-62.1
C2	O2	C1	H1C	177.9
C1	O2	C2	O1	1.4(2)
C1	O2	C2	C3	-178.6(1)
C5	O3	C4	C3	180.0(1)
C5	O3	C4	H4	0
C4	O3	C5	H5	-2.5
C4	O3	C5	C6	177.5(1)
C8	O5	C7	O4	1.2(2)
C8	O5	C7	C6	-178.8(1)
C7	O5	C8	H8A	-63.6
C7	O5	C8	H8B	176.4
C7	O5	C8	H8C	56.4
O1	C2	C3	H3	-175.1
O1	C2	C3	C4	4.9(2)
O2	C2	C3	H3	4.9
O2	C2	C3	C4	-175.1(1)
C2	C3	C4	O3	179.8(1)
C2	C3	C4	H4	-0.2
H3	C3	C4	O3	-0.2
H3	C3	C4	H4	179.9
O3	C5	C6	H6	-0.3
O3	C5	C6	C7	179.7(1)
H5	C5	C6	H6	179.7
H5	C5	C6	C7	-0.3
C5	C6	C7	O4	-3.2(2)
C5	C6	C7	O5	176.8(1)
H6	C6	C7	O4	176.8
H6	C6	C7	O5	-3.2

Supplementary Table 19. Bond Lengths for *EE*-OBBO.

Atom1	Atom2	Length/Å
O001	C005	1.364(2)
O001	C008	1.367(2)
O002	C004	1.228(2)
O003	C007	1.218(2)
C004	C006	1.461(2)
C004	C00B	1.501(3)
C005	H005	0.93
C005	C006	1.323(2)
C006	H006	0.93
C007	C009	1.499(2)

C007	C00A	1.467(2)
C008	H008	0.93
C008	C00A	1.326(2)
C009	H00A	0.96
C009	H00B	0.96
C009	H00C	0.96
C00A	H00D	0.93
C00B	H00E	0.96
C00B	H00F	0.96
C00B	H00G	0.96

Supplementary Table 20. Bond Angles for *EE*-OBBO.

Atom1	Atom2	Atom3	Angle/°
C005	O001	C008	117.9(1)
O002	C004	C006	119.5(2)
O002	C004	C00B	120.2(2)
C006	C004	C00B	120.2(1)
O001	C005	H005	120.5
O001	C005	C006	119.0(1)
H005	C005	C006	120.5
C004	C006	C005	124.3(2)
C004	C006	H006	117.8
C005	C006	H006	117.8
O003	C007	C009	121.7(2)
O003	C007	C00A	123.0(2)
C009	C007	C00A	115.2(1)
O001	C008	H008	120.3
O001	C008	C00A	119.5(2)
H008	C008	C00A	120.3
C007	C009	H00A	109.5
C007	C009	H00B	109.5
C007	C009	H00C	109.4
H00A	C009	H00B	109.5
H00A	C009	H00C	109.5
H00B	C009	H00C	109.5
C007	C00A	C008	121.2(2)
C007	C00A	H00D	119.4
C008	C00A	H00D	119.4
C004	C00B	H00E	109.5
C004	C00B	H00F	109.5
C004	C00B	H00G	109.5
H00E	C00B	H00F	109.5
H00E	C00B	H00G	109.5

H00F	C00B	H00G	109.5

Supplementary Table 21. Torsion Angles for *EE*-OBBO.

Atom1	Atom2	Atom3	Atom4	Torsion angles/°
C008	O001	C005	H005	-5.6
C008	O001	C005	C006	174.4(1)
C005	O001	C008	H008	1.9
C005	O001	C008	C00A	-178.1(2)
O002	C004	C006	C005	176.0(2)
O002	C004	C006	H006	-4
C00B	C004	C006	C005	-6.1(3)
C00B	C004	C006	H006	173.9
O002	C004	C00B	H00E	-38.3
O002	C004	C00B	H00F	-158.3
O002	C004	C00B	H00G	81.7
C006	C004	C00B	H00E	143.8
C006	C004	C00B	H00F	23.9
C006	C004	C00B	H00G	-96.2
O001	C005	C006	C004	178.3(1)
O001	C005	C006	H006	-1.7
H005	C005	C006	C004	-1.7
H005	C005	C006	H006	178.3
O003	C007	C009	H00A	-118.1
O003	C007	C009	H00B	121.9
O003	C007	C009	H00C	1.9
C00A	C007	C009	H00A	61.6
C00A	C007	C009	H00B	-58.4
C00A	C007	C009	H00C	-178.4
O003	C007	C00A	C008	6.1(3)
O003	C007	C00A	H00D	-174
C009	C007	C00A	C008	-173.7(2)
C009	C007	C00A	H00D	6.3
O001	C008	C00A	C007	177.8(1)
O001	C008	C00A	H00D	-2.2
H008	C008	C00A	C007	-2.2
H008	C008	C00A	H00D	177.8
